# Supplementary material for: Emergent universal long-range structure in random-organizing systems
Source: Nat Commun. 2026 Jan 23;17:2346. doi: 10.1038/s41467-026-68601-2 (PMC12979774; doi:10.1038/s41467-026-68601-2)
Supplement: Supplementary file 1 — Supplementary Information [file 41467_2026_68601_MOESM1_ESM.pdf]

Supplementary Information

Emergent universal long-range structure in random-organizing systems

Satyam Anand,[1, 2, *] Guanming Zhang,[2, 3, †] and Stefano Martiniani[1, 2, 3, 4, ‡]

[1] *Courant Institute of Mathematical Sciences, New York University, New York, NY 10003, USA*
[2] *Center for Soft Matter Research, Department of Physics, New York University, New York, NY 10003, USA*
[3] *Simons Center for Computational Physical Chemistry, Department of Chemistry, New York University, New York, NY 10003, USA*
[4] *Center for Neural Science, New York University, New York, NY 10003, USA*

CONTENTS

I. Theory 1
A. Continuous-time approximation of discrete-time dynamics 1
B. Fluctuating hydrodynamics 4
1. Fokker-Planck method 4
2. Dean's method 6
C. Relationship between emergent structure and flatness of the energy landscape in SGD 13
D. Effect of thermal noise 16

References 16

I. THEORY

A. Continuous-time approximation of discrete-time dynamics

Random-organizing systems are discrete-time systems (Eqs. 1, 2, and 3 in main text). Following the framework of stochastic modified equations [1, 2], we first approximate these systems by a continuous-time dynamics. Consider a generic discrete-time evolution equation,

$$\begin{aligned}\mathbf{x}^{m+1} &= \mathbf{x}^m + \boldsymbol{\vartheta} \\ &= \mathbf{x}^m + \langle\boldsymbol{\vartheta}\rangle + (\boldsymbol{\vartheta} - \langle\boldsymbol{\vartheta}\rangle)\,,\end{aligned} \tag{S1}$$

where $\mathbf{x}^m$ is the value of variable $\mathbf{x}$ at time-step m, $\boldsymbol{\vartheta}$ is an arbitrary noise with mean $\langle\vartheta_\alpha\rangle$ and covariance matrix $\langle(\vartheta_\alpha - \langle\vartheta_\alpha\rangle)(\vartheta_\beta - \langle\vartheta_\beta\rangle)\rangle$, and $\langle.\rangle$ denotes the expectation value. We approximate $(\boldsymbol{\vartheta} - \langle\boldsymbol{\vartheta}\rangle)$ in Eq. S1 by a Gaussian noise $\sqrt{\langle(\boldsymbol{\vartheta} - \langle\boldsymbol{\vartheta}\rangle)(\boldsymbol{\vartheta} - \langle\boldsymbol{\vartheta}\rangle)^\intercal\rangle}\cdot\boldsymbol{\varrho}$, where $\boldsymbol{\varrho}$ is a Gaussian noise with $\langle\varrho_\alpha\rangle = 0$ and $\langle\varrho_\alpha\varrho_\beta\rangle = \delta_{\alpha\beta}$ to get,

$$\mathbf{x}^{m+1} = \mathbf{x}^m + \langle\boldsymbol{\vartheta}\rangle + \sqrt{\langle(\boldsymbol{\vartheta} - \langle\boldsymbol{\vartheta}\rangle)(\boldsymbol{\vartheta} - \langle\boldsymbol{\vartheta}\rangle)^\intercal\rangle}\cdot\boldsymbol{\varrho}, \tag{S2}$$

where $\sqrt{.}$ is the matrix square root. We assume the characteristic time of one discrete time-step to be τ, giving time elapsed after m discrete time-steps as $t = m\tau$. We can now rewrite Eq. S2 as,

$$\mathbf{x}^{(m+1)\tau} = \mathbf{x}^{m\tau} + \tau\left(\frac{\langle\boldsymbol{\vartheta}\rangle}{\tau}\right) + \sqrt{\tau}\left(\frac{\sqrt{\langle(\boldsymbol{\vartheta} - \langle\boldsymbol{\vartheta}\rangle)(\boldsymbol{\vartheta} - \langle\boldsymbol{\vartheta}\rangle)^\intercal\rangle}}{\sqrt{\tau}}\right)\cdot\boldsymbol{\varrho}. \tag{S3}$$

Eq. S3 is nothing but the Euler-Maruyama discretization of a continuous-time stochastic differential equation (SDE) given by,

$$\frac{d\mathbf{x}(t)}{dt} = \underbrace{\frac{\langle\boldsymbol{\vartheta}\rangle}{\tau}}_{\text{deterministic term}} + \underbrace{\frac{\sqrt{\langle(\boldsymbol{\vartheta} - \langle\boldsymbol{\vartheta}\rangle)(\boldsymbol{\vartheta} - \langle\boldsymbol{\vartheta}\rangle)^\intercal\rangle}}{\sqrt{\tau}}\cdot\boldsymbol{\varphi}(t)}_{\text{noise term}}, \tag{S4}$$

* Equal contribution; sa7483@nyu.edu
† Equal contribution; gz2241@nyu.edu
‡ sm7683@nyu.edu

where $\boldsymbol{\varphi}(t)$ is a Gaussian noise with mean $\langle \varphi_\alpha(t) \rangle = 0$, and covariance matrix $\langle \varphi_\alpha(t)\varphi_\beta(t) \rangle = \delta_{\alpha\beta}\delta(t-t')$. Thus, Eq. S4 is a continuous-time approximation of the discrete-time Eq. S1. We now apply this generic procedure to RO, BRO, and SGD.

In RO, the dynamics of the position of particle i at time-step $m+1$ ($\mathbf{x}_i^{m+1}$) is given by,

$$\mathbf{x}_i^{m+1} = \mathbf{x}_i^m + \epsilon \sum_{j\in\Gamma_i^m} u_{ji}^m \boldsymbol{\zeta}_{ji}^m, \tag{S5}$$

where ϵ controls the magnitude of the pairwise kick given by particle j to i, u_{ji}^m is a random number sampled from a standard uniform distribution ($U[0,1]$) at time-step m, $\boldsymbol{\zeta}_{ji}^m$ is a random unit vector sampled uniformly on the surface of a d-dimensional unit hypersphere at time-step m, and $\Gamma_i^m = \{j \mid |\mathbf{x}_j^m - \mathbf{x}_i^m| < 2R, j \neq i\}$ is the set containing all particles that overlap with particle i at time-step m. The covariance matrix of the complete noise vector $\boldsymbol{\omega}_{ij}^m = \epsilon u_{ij}^m \boldsymbol{\zeta}_{ij}^m$ is $\mathrm{cov}[\omega_{ij,\alpha}^m \omega_{kl,\beta}^n] = (\epsilon^2/3d)\,\delta^{mn}\delta_{\alpha\beta}(\delta_{ik}\delta_{jl} + c\,\delta_{il}\delta_{jk})$, where c is the Pearson correlation coefficient between $\omega_{ij,\alpha}^m$ and $\omega_{ji,\alpha}^m$. Comparing Eq. S5 with Eq. S1 to get $\boldsymbol{\vartheta} = \epsilon\sum_{j\in\Gamma_i^m} u_{ji}^m \boldsymbol{\zeta}_{ji}^m$, we can write the SDE for RO as,

$$\frac{d\mathbf{x}_i(t)}{dt} = \underbrace{\sqrt{\frac{\epsilon^2}{3d\tau}} \sum_{j=1}^{N} \sqrt{\mathbf{1}_{(0,2R)}(r_{ij})\mathbf{I}} \cdot \boldsymbol{\xi}_{ji}(t)}_{\text{noise term}}, \tag{S6}$$

where $\mathbf{1}_{(0,2R)}(r_{ij})$ is an indicator function such that $\mathbf{1}_{(0,2R)}(r_{ij}) = 1\ \forall\ r_{ij} \in (0,2R)$ and $\mathbf{1}_{(0,2R)}(r_{ij}) = 0$ otherwise, $r_{ij} = |\mathbf{x}_j^m - \mathbf{x}_i^m|$, $\mathbf{I}$ is the identity matrix, $\boldsymbol{\xi}_{ji}$ is a pairwise, Gaussian noise given by the particle j to particle i having mean $\langle \xi_{ji,\alpha}(t) \rangle = 0$ and covariance matrix $\langle \xi_{ij,\alpha}(t)\,\xi_{kl,\beta}(t') \rangle = \delta(t-t')\,\delta_{\alpha\beta}(\delta_{ik}\delta_{jl} + c\,\delta_{il}\delta_{jk})$, where c is the Pearson correlation coefficient between $\xi_{ij,\alpha}(t)$ and $\xi_{ji,\alpha}(t)$.

In BRO, the dynamics of the position of particle i at time-step $m+1$ ($\mathbf{x}_i^{m+1}$) is given by,

$$\mathbf{x}_i^{m+1} = \mathbf{x}_i^m + \epsilon \sum_{j\in\Gamma_i^m} u_{ji}^m \hat{\mathbf{x}}_{ji}^m, \tag{S7}$$

where ϵ controls the magnitude of the pairwise kick given by particle j to i, u_{ji}^m is a random number sampled from a standard uniform distribution ($U[0,1]$) at time-step m, and $\hat{\mathbf{x}}_{ji}^m = -(\mathbf{x}_j^m - \mathbf{x}_i^m)/|\mathbf{x}_j^m - \mathbf{x}_i^m|$ is the deterministic unit vector pointing from the center of particle j to i at time-step m. The covariance of the complete noise vector $\boldsymbol{\omega}_{ij}^m = \epsilon u_{ij}^m \hat{\mathbf{x}}_{ij}^m$ is $\mathrm{cov}[\omega_{ij,\alpha}^m \omega_{kl,\beta}^n] = (\epsilon^2/12)\,\delta^{mn}\hat{x}_{ji,\alpha}^n \hat{x}_{ji,\beta}^n(\delta_{ik}\delta_{jl} + c\,\delta_{il}\delta_{jk})$, where c is the Pearson correlation coefficient between $\omega_{ij,\alpha}^m$ and $\omega_{ji,\alpha}^m$. Comparing Eq. S7 with Eq. S1 to get $\boldsymbol{\vartheta} = \epsilon\sum_{j\in\Gamma_i^m} u_{ji}^m \hat{\mathbf{x}}_{ji}^m$, we can write the SDE for BRO as,

$$\frac{d\mathbf{x}_i(t)}{dt} = \underbrace{-\frac{\epsilon R}{\mathcal{E}\tau} \sum_{j=1}^{N} \nabla_i V_{ji}}_{\text{deterministic term}} + \underbrace{\sqrt{\frac{\epsilon^2 R^2}{3\tau\mathcal{E}^2}} \sum_{j=1}^{N} \sqrt{\nabla_i V_{ji} \nabla_i V_{ji}^{\intercal}} \cdot \boldsymbol{\xi}_{ji}(t)}_{\text{noise term}}, \tag{S8}$$

where $\nabla_i = \nabla_{\mathbf{x}_i}$, and V_{ji} is a pairwise, linear, repulsive potential given by

$$V_{ij}(r) = \begin{cases} \mathcal{E}\left(1 - \frac{r_{ij}}{2R}\right), & \text{if } 0 < r_{ij} < 2R, \\ 0, & \text{otherwise,} \end{cases} \tag{S9}$$

where $\mathcal{E}$ is the characteristic energy scale.

In SGD, we consider particles interacting via a pairwise potential. The dynamics of the position of particle i at time-step $m+1$ ($\mathbf{x}_i^{m+1}$) is given by,

$$\mathbf{x}_i^{m+1} = \mathbf{x}_i^m - \alpha \sum_{j\in\Gamma_i^m} \theta_{ji}^m \nabla_i V_{ji}^m \tag{S10}$$

where α is the learning rate, $V_{ji}^m = V(|\mathbf{x}_j^m - \mathbf{x}_i^m|)$ is the interaction potential, and θ_{ji}^m is a random number sampled from a Bernoulli distribution having parameter b_f (batch fraction) at time-step m. The covariance of the complete noise vector $\boldsymbol{\omega}_{ij}^m = -\alpha\theta_{ij}^m \nabla_i V_{ij}^m$ is $\mathrm{cov}[\omega_{ij,\alpha}^m \omega_{kl,\beta}^n] = \alpha^2 b_f(1-b_f)\,\delta^{mn}\partial_{i,\alpha}V_{ji}^m\,\partial_{i,\beta}V_{ji}^n(\delta_{ik}\delta_{jl} + c\,\delta_{il}\delta_{jk})$, where c is the Pearson correlation coefficient between $\omega_{ij,\alpha}^m$ and $\omega_{ji,\alpha}^m$. While V_{ji} can be any pairwise potential in SGD, either short- or long-range, here, we consider a class of short-range, repulsive potentials given by

$$V_{ij}(r) = \begin{cases} \frac{\mathcal{E}}{p}\left(1 - \frac{r_{ij}}{2R}\right)^p, & \text{if } 0 < r_{ij} < 2R, \\ 0, & \text{otherwise,} \end{cases} \tag{S11}$$

where $\mathcal{E}$ is the characteristic energy scale, and p controls the stiffness of the potential. Comparing Eq. S10 with Eq. S1 to get $\boldsymbol{\vartheta} = -\theta_{ij}^m \alpha \nabla_i V_{ji}^m$, we can write the SDE for SGD as,

$$\frac{d\mathbf{x}_i(t)}{dt} = \underbrace{-\frac{\alpha b_f}{\tau}\sum_{j=1}^{N}\nabla_i V_{ji}}_{\text{deterministic term}} + \underbrace{\sqrt{\frac{\alpha^2 b_f(1-b_f)}{\tau}}\sum_{j=1}^{N}\sqrt{\nabla_i V_{ji}\nabla_i V_{ji}^{\intercal}}\cdot\boldsymbol{\xi}_{ji}(t)}_{\text{noise term}}\,. \tag{S12}$$

The SDE for RO, BRO, and SGD (Eqs. S6, S8, and S12) can be generically written as an overdamped Langevin equation of the form

$$\frac{d\mathbf{x}_i(t)}{dt} = \underbrace{-\frac{1}{\gamma}\sum_{j=1}^{N}\nabla_i V_{ji}}_{\text{deterministic term}} + \underbrace{\sum_{j=1}^{N}\sqrt{\boldsymbol{\Lambda}_{ji}}\cdot\boldsymbol{\xi}_{ji}}_{\text{noise term}}, \tag{S13}$$

where γ is the friction constant, V_{ji}, $\boldsymbol{\Lambda}_{ji}$ are short-range, pairwise interaction potential and diffusion matrix between particles j and i, respectively. $\boldsymbol{\xi}_{ji}$ is a pairwise, Gaussian noise given by particle j to particle i with mean $\langle \xi_{ji,\alpha}(t)\rangle = 0$ and covariance matrix $\langle \xi_{ij,\alpha}(t)\,\xi_{kl,\beta}(t')\rangle = \delta(t-t')\,\delta_{\alpha\beta}(\delta_{ik}\delta_{jl} + c\,\delta_{il}\delta_{jk})$, where c is the Pearson correlation coefficient between $\xi_{ij,\alpha}(t)$ and $\xi_{ji,\alpha}(t)$. γ, V_{ji}, and $\boldsymbol{\Lambda}_{ji}$ take different values and functional forms for different systems (Table S1, Eqs. S6, S8, and S12). $V_{ii} = \Lambda_{ii,\alpha\beta} = 0$ for BRO, RO and SGD due to the absence of self-interaction in all three systems. Further, for all three systems, both V_{ij} and $\boldsymbol{\Lambda}_{ij}$ are invariant under particle exchange, i.e., $V_{ij} = V_{ji}$ and $\boldsymbol{\Lambda}_{ij} = \boldsymbol{\Lambda}_{ji}$, and $\boldsymbol{\Lambda}_{ij}$ is a symmetric matrix ($\Lambda_{ij,\alpha\beta} = \Lambda_{ij,\beta\alpha}$).

	Friction coefficient (γ)	Interaction potential (V_{ji})	Diffusion matrix ($\boldsymbol{\Lambda}_{ji}$)
RO	—	0	$\frac{\epsilon^2}{3d\tau}\mathbf{1}_{(0,2R)}(r_{ij})\mathbf{I}$
BRO	$\frac{\mathcal{E}\tau}{\epsilon R}$	$V_{ij}(r) = \begin{cases}\mathcal{E}\left(1-\frac{r_{ij}}{2R}\right), & \text{if } 0<r_{ij}<2R,\\ 0, & \text{otherwise}\end{cases}$	$\frac{\epsilon^2 R^2}{3\tau\mathcal{E}^2}\nabla_i V_{ji}\nabla_i V_{ji}^{\intercal}$
SGD	$\frac{\tau}{\alpha b_f}$	$V_{ij}(r) = \begin{cases}\frac{\mathcal{E}}{p}\left(1-\frac{r_{ij}}{2R}\right)^p, & \text{if } 0<r_{ij}<2R,\\ 0, & \text{otherwise}\end{cases}$	$\frac{\alpha^2 b_f(1-b_f)}{\tau}\nabla_i V_{ji}\nabla_i V_{ji}^{\intercal}$

TABLE S1. Parameters for the continuous-time generalized model of random-organizing systems.

Notice that for RO, the deterministic (first) term in Eq. S13 vanishes, and the noise (second) term is isotropic—consistent with the fact that pairwise kicks are randomly directed in RO (Eqs. S5, and S6). The noise term in BRO, however, is anisotropic—directed along the line joining the center of particles (Eq. S8). The noise term in SGD is also anisotropic and directed along the line joining the center of particles—in fact, SGD with a linear potential ($p = 1$ in Eq. S11) maps exactly to BRO (except for prefactors) (Eqs. S8, and S12) [2]. Finally, it is worth noting that the noise in discrete-time SGD is on the selection of particles, while in the SDE, it appears as an interaction noise (Eqs. S10, and S12). All SDEs are interpreted in the Itô sense.

For an overdamped system of interacting particles at thermal equilibrium, the noise is additive, and uncorrelated across particles. In contrast, the noise in Eq. S13 is multiplicative and pairwise correlated between particles. This distinction provides a clear microscopic explanation for why RO, BRO, and SGD dynamics are intrinsically out of equilibrium.

Energy evolution in SGD. Optimization methods such as gradient flow minimize the total energy. SGD, however, is different from gradient flow because of (i) a finite learning rate, and (ii) batch selection [3]. Thus, SGD does not minimize the total energy, rather the partial energy. Here, we show the evolution of energy for Eq. S13. The total energy E is,

$$E = \frac{1}{2}\sum_i\sum_{j\neq i} V(\mathbf{x}_i, \mathbf{x}_j). \tag{S14}$$

The variation in E can then be written as (up to second order in $\Delta\mathbf{x}_i$),

$$\begin{aligned} dE &= \frac{1}{2}\sum_i \sum_{j\neq i} \left[V(\mathbf{x}_i + \Delta\mathbf{x}_i, \mathbf{x}_j + \Delta\mathbf{x}_j) - V(\mathbf{x}_i, \mathbf{x}_j)\right] \\ &= \frac{1}{2}\sum_i \sum_{j\neq i} \left[\Delta\mathbf{x}_i^T \nabla_{\mathbf{x}_i} V_{ij} + \Delta\mathbf{x}_j^T \nabla_{\mathbf{x}_j} V_{ij} + \frac{1}{2}\Delta\mathbf{x}_i^T \nabla_{\mathbf{x}_i}\nabla_{\mathbf{x}_i} V_{ij}\Delta\mathbf{x}_i + \frac{1}{2}\Delta\mathbf{x}_j^T \nabla_{\mathbf{x}_j}\nabla_{\mathbf{x}_j} V_{ij}\Delta\mathbf{x}_j + \Delta\mathbf{x}_i^T \nabla_{\mathbf{x}_i}\nabla_{\mathbf{x}_j} V_{ij}\Delta\mathbf{x}_j\right]. \end{aligned} \tag{S15}$$

We know that $\langle \xi_{ij,\alpha}(t)\, \xi_{kl,\beta}(t')\rangle = \delta(t-t')\,\delta_{\alpha\beta}(\delta_{ik}\delta_{jl} + c\,\delta_{il}\delta_{jk})$, and $\Delta\mathbf{x}_i = \mathbf{x}_i(t+\Delta t) - \mathbf{x}_i(t)$ evolves according to Eq. S13. We can then write the following equations under the Itô interpretation,

$$\begin{aligned} \langle \Delta\mathbf{x}_i\rangle &= -\frac{1}{\gamma}\sum_{j\neq i} \nabla_{\mathbf{x}_i} V_{ij}\Delta t, \\ \langle \Delta\mathbf{x}_i \Delta\mathbf{x}_j^T\rangle &= \delta_{ij}\sum_{k\neq i} \mathbf{\Lambda}_{jk}\Delta t + c\,\mathbf{\Lambda}_{ij}\Delta t, \end{aligned} \tag{S16}$$

where terms of order higher than Δt are neglected. Note that $\mathbf{\Lambda}_{ii,\alpha\beta} = 0$ (no self-interaction). Using Eqs. S15 and S16, we now get,

$$\frac{d\langle E\rangle}{dt} = -\frac{1}{\gamma}\sum_i \left|\sum_{j\neq i} \nabla_{\mathbf{x}_i} V_{ij}\right|^2 + \frac{1}{2}\sum_i\sum_{j\neq i} \mathrm{Tr}\left[\nabla_{\mathbf{x}_i}\nabla_{\mathbf{x}_i} V_{ij}\left(\sum_{k\neq i}\mathbf{\Lambda}_{ki}\right)\right] + \frac{c}{2}\sum_i\sum_{j\neq i}\mathrm{Tr}\left[\nabla_{\mathbf{x}_i}\nabla_{\mathbf{x}_j} V_{ij}\left(\mathbf{\Lambda}_{ij}\right)\right]. \tag{S17}$$

The first term in Eq. S17 always minimizes the total energy, whereas the second and third terms do not. Hence, the total energy is not strictly minimized. However, energy minima are a fixed point of the equation since the right hand side of the evolution equation vanishes, i.e., $\frac{d\mathbf{x}_i}{dt} = 0$, when $\nabla_{\mathbf{x}_i}\sum_{j\neq i} V(\mathbf{x}_i, \mathbf{x}_j) = 0$ for each particle i. This behavior mirrors SGD dynamics in optimization, where stochastic batch selection causes SGD to minimize not the full loss (or energy) but a fluctuating partial loss at each step, and where minima with vanishing gradients across all batches act as fixed points of the dynamics. In the regime of weak, perturbative noise (e.g., batch fraction $b_f \sim 1$), the diffusion matrix is negligible compared to the drift term. As a result, the system primarily follows the gradient-descent drift, and relaxes toward an energy minimum.

B. Fluctuating hydrodynamics

Starting from the continuous-time description of random-organizing systems (Eq. S13), we now coarse-grain the microscopic dynamics to get a continuum description.

1. Fokker-Planck method

Defining the N-particle density as $\rho_N(\{\mathbf{x}_N\}, t)$, where $\{\mathbf{x}_N\} \equiv \{\mathbf{x}_1, \mathbf{x}_2, ..., \mathbf{x}_N\}$, the dynamics of $\rho_N(\{\mathbf{x}_N\}, t)$ is given by the Fokker-Planck equation,

$$\frac{\partial \rho_N(\{\mathbf{x}_N\}, t)}{\partial t}\Delta t = -\sum_i \partial_{x_{i,\alpha}}(\langle \Delta x_{i,\alpha}\rangle \rho_N) + \frac{1}{2}\sum_i\sum_j \partial_{x_{i,\alpha}}\partial_{x_{j,\beta}}(\langle \Delta x_{i,\alpha}\Delta x_{j,\beta}\rangle \rho_N), \tag{S18}$$

where $\Delta x_{i,\alpha} = x_{i,\alpha}(t+\Delta t) - x_{i,\alpha}(t)$. Using Ito calculus, we evaluate $\langle \Delta x_{i,\alpha}\rangle$ and $\langle \Delta x_{i,\alpha}\Delta x_{j,\beta}\rangle$ to get,

$$\begin{aligned} \langle \Delta x_{i,\alpha}\rangle &= -\frac{1}{\gamma}\partial_{x_{i,\alpha}}\left(\sum_j V_{ji}\right)\Delta t + \mathcal{O}(\Delta t^{3/2}), \\ \langle \Delta x_{i,\alpha}\Delta x_{j,\beta}\rangle &= \delta_{ij}\sum_k \Lambda_{ki,\alpha\beta}(t)\Delta t + c\Lambda_{ij,\alpha\beta}(t)\Delta t + \mathcal{O}(\Delta t^{3/2}). \end{aligned} \tag{S19}$$

Finally, after neglecting terms of order higher than $\mathcal{O}(\Delta t)$, we get,

$$\frac{\partial \rho_N(\{\mathbf{x}_N\},t)}{\partial t} = \frac{1}{\gamma}\sum_i \partial_{x_i,\alpha}\left[\left(\partial_{x_i,\alpha}\sum_j V_{ji}\right)\rho_N\right] + \frac{1}{2}\sum_i\sum_j \partial_{x_i,\alpha}\partial_{x_j,\beta}\left[\left(\delta_{ij}\sum_k \Lambda_{ki,\alpha\beta}(t) + c\Lambda_{ij,\alpha\beta}\right)\rho_N\right]. \tag{S20}$$

Notice that the n-particle density $\rho_n(\mathbf{x}_1, \mathbf{x}_2, ..., \mathbf{x}_n)$ is obtained by integrating the N-particle density ρ_N over the remaining $N-n$ particle coordinates: $\rho_n(\mathbf{x}_1, \mathbf{x}_2, ..., \mathbf{x}_n) = \frac{N!}{(N-n)!}\int \rho_N(\mathbf{x}_1, \mathbf{x}_2, ..., \mathbf{x}_N)\, d\mathbf{x}_{n+1}d\mathbf{x}_{n+2}...d\mathbf{x}_N$. Integrating Eq. S20 over particle coordinates yields a hierarchy of coupled equations analogous to the BBGKY hierarchy in kinetic theory, where the equation for ρ_n depends on ρ_{n+1} [4]. The first equation in this hierarchy, governing the one-particle density $\rho_1(\mathbf{x}_1)$, is given by,

$$\frac{\partial \rho_1(\mathbf{x}_1,t)}{\partial t} = \frac{1}{\gamma}\partial_{x_1,\alpha}\int \partial_{x_1,\alpha}(V_{21})\rho_2(\mathbf{x}_1,\mathbf{x}_2)d\mathbf{x}_2 + \frac{1}{2}\partial_{x_1,\alpha}\partial_{x_1,\beta}\int \Lambda_{12,\alpha\beta}\ \rho_2(\mathbf{x}_1,\mathbf{x}_2)d\mathbf{x}_2, \tag{S21}$$

where we have used the fact that the boundary terms vanish, and $\Lambda_{ii} = 0$ (no self-interactions). We now use the mean-field closure approximation $\rho_2(\mathbf{x}_1,\mathbf{x}_2) = \rho_1(\mathbf{x}_1)\rho_1(\mathbf{x}_2)$ on Eq. S21 to get,

$$\frac{\partial \rho_1(\mathbf{x}_1,t)}{\partial t} = \frac{1}{\gamma}\partial_{x_1,\alpha}\left(\rho_1(\mathbf{x}_1)\int \partial_{x_1,\alpha}(V_{21})\rho_1(\mathbf{x}_2)d\mathbf{x}_2\right) + \frac{1}{2}\partial_{x_1,\alpha}\partial_{x_1,\beta}\left(\rho_1(\mathbf{x}_1)\int \Lambda_{12,\alpha\beta}\ \rho_1(\mathbf{x}_2)d\mathbf{x}_2\right). \tag{S22}$$

Since all particles are identical and particle indices 1 and 2 are arbitrary, we replace $\mathbf{x}_1 \to \mathbf{x}$ and $\mathbf{x}_2 \to \mathbf{y}$ to get,

$$\frac{\partial \rho(\mathbf{x},t)}{\partial t} = \frac{1}{\gamma}\partial_{x,\alpha}\left(\rho(\mathbf{x})\int \rho(\mathbf{y})\ \partial_{x,\alpha}V(\mathbf{x},\mathbf{y})d\mathbf{y}\right) + \frac{1}{2}\partial_{x,\alpha}\partial_{x,\beta}\left(\rho(\mathbf{x})\int \Lambda(\mathbf{x},\mathbf{y})_{\alpha\beta}\ \rho(\mathbf{y})d\mathbf{y}\right). \tag{S23}$$

Eq. S23 is the Fokker-Planck equation describing the coarse-grained density evolution for all random-organizing systems.

Linearization. To gain further analytical insights, we now linearize the density to first order around a spatiotemporally constant mean density $\bar{\rho}$ as $\rho(\mathbf{x},t) = \bar{\rho} + \delta\rho(\mathbf{x},t)$. Plugging this into Eq. S23, and retaining terms up to the first order, we get the evolution of density fluctuation $\delta\rho(\mathbf{x},t)$ as,

$$\frac{\partial \delta\rho(\mathbf{x},t)}{\partial t} = \frac{\bar{\rho}}{\gamma}\ \partial_\alpha\left(\int \delta\rho(\mathbf{y},t)\partial_\alpha V(\mathbf{x}-\mathbf{y})\, d\mathbf{y}\right) + \frac{\bar{\rho}}{2}\partial_\alpha\partial_\beta\left(\int \delta\rho(\mathbf{y},t)\Lambda(\mathbf{x}-\mathbf{y})_{\alpha\beta}\, d\mathbf{y} + \delta\rho(\mathbf{x},t)\int \Lambda(\mathbf{x}-\mathbf{y})_{\alpha\beta}\, d\mathbf{y}\right). \tag{S24}$$

Defining the Fourier transform in space and time for any function $f(\mathbf{x},t)$ as $\hat{f}(\mathbf{k},\omega) = \int\int f(\mathbf{x},t)e^{-i\mathbf{k}\cdot\mathbf{x}}e^{-i\omega t}d\mathbf{x}dt$, and taking Fourier transform of Eq. S24, we get,

$$i\omega\hat{\delta\rho}(\mathbf{k},\omega) = -\frac{\bar{\rho}}{\gamma}|\mathbf{k}|^2\hat{\delta\rho}(\mathbf{k},\omega)\hat{V}(\mathbf{k}) - \frac{\bar{\rho}}{2}\hat{\delta\rho}(\mathbf{k},\omega)k_\alpha k_\beta\left(\hat{\Lambda}(\mathbf{k})_{\alpha\beta} + A_{\alpha\beta}\right), \tag{S25}$$

where $A_{\alpha\beta} = \int \Lambda(\mathbf{r})_{\alpha\beta}\, d\mathbf{r}$. Rearranging Eq. S25, we get the trivial solution,

$$\hat{\delta\rho}(\mathbf{k},\omega) = 0. \tag{S26}$$

Using Eq. S26, we now write the dynamic structure factor $S(\mathbf{k},\omega)$ as,

$$S(\mathbf{k},\omega) = \frac{1}{2\pi TN}\left\langle \hat{\delta\rho}(\mathbf{k},\omega)\hat{\delta\rho}^*(\mathbf{k},\omega)\right\rangle = 0 \tag{S27}$$

where $\int dt = T$ is the total observation time, and $N = \int \rho(\mathbf{x},t)\, d\mathbf{x}$ is the total number of particles. We now write the static structure factor $S(\mathbf{k})$ as,

$$S(\mathbf{k}) = \int_{-\infty}^{\infty} S(\mathbf{k},\omega)\, d\omega = 0. \tag{S28}$$

It is evident from Eq. S26 that at steady-state, $\rho(\mathbf{x},t) = \bar{\rho}$, i.e., a spatiotemporally constant density. This leads to the trivial result $S(\mathbf{k}) = 0$, which is inconsistent with particle simulations (Fig. 2 in the main text). Thus, the widely used Fokker-Planck method is not sufficient to describe the density evolution and long-range structure for random-organizing systems. This motivates us to use a method which can retain an important feature of our system—the pairwise noise correlation between particles—even at the coarse-grained level.

Steady-state distribution. Here we address the question of whether the steady-state solution for the evolution of the N-particle density ρ_N is given by the Boltzmann distribution, as for interacting Brownian particles at equilibrium [5, 6] (Eq. S20). Plugging the steady-state distribution ρ_N^s in Eq. S20 and noting that $\partial\rho_N^s/\partial t = 0$, we get,

$$\frac{1}{\gamma}\sum_i \partial_{x_i,\alpha}\left[\left(\partial_{x_i,\alpha}\sum_j V_{ji}\right)\rho_N^s\right] + \frac{1}{2}\sum_i\sum_j \partial_{x_i,\alpha}\partial_{x_j,\beta}\left[\left(\delta_{ij}\sum_k \Lambda_{ki,\alpha\beta}(t) + c\Lambda_{ij,\alpha\beta}\right)\rho_N^s\right] = 0. \tag{S29}$$

Notice that Eq. S29 is difficult to solve for arbitrary $\Lambda_{ij,\alpha\beta}$ and noise correlation c. However, substituting $\Lambda_{ij,\alpha\beta} = a\delta_{\alpha\beta}$, and $c = 0$ in Eq. S29, where a is some constant, we get after some algebra,

$$\sum_i \partial_{x_i,\alpha}\left[\frac{1}{\gamma}\left(\partial_{x_i,\alpha}U_i\right)\rho_N^s + \frac{Na}{2}\partial_{x_i,\alpha}\rho_N^s\right] = 0, \tag{S30}$$

where $U_i = \sum_j V_{ij}$, and N is the number of particles. The solution of Eq. S30 is given by the Boltzmann distribution $\rho_N^B = (1/Z)e^{-(2/Na\gamma)E}$, where $E = \sum_i\sum_{j\geq i} V_{ij}$, and Z is the normalization factor. This is not surprising since substituting $\Lambda_{ij,\alpha\beta} = a\delta_{\alpha\beta}$ and $c = 0$ in Eq. S13 reduces it to a system of interacting Brownian particles at equilibrium, whose steady-state solution is known to be the Boltzmann distribution [5, 6]. Notice, however, that since $\Lambda_{ij,\alpha\beta} \neq a\delta_{\alpha\beta}$ and $c \neq 0$, in general, for random-organizing systems (Table S1), the Boltzmann distribution with a time-independent temperature (as given by ρ_N^B) is not the steady-state solution for Eq. S29.

2. Dean's method

We now use Dean's method to reach a coarse-grained description. Dean's method was first introduced to systematically coarse-grain an equilibrium system of interacting Brownian particles, starting from an overdamped Langevin equation with an additive white noise term [7]. This method has since been adapted to study systems having multiplicative noise terms [8–10]. Here, we extend this method to account for multiplicative noise which is also pairwise correlated between particles.

We define the density function for a single particle $\rho_i(\mathbf{x}, t)$ as

$$\rho_i(\mathbf{x}, t) = \delta(\mathbf{x}_i(t) - \mathbf{x}(t)). \tag{S31}$$

The global density $\rho(\mathbf{x}, t)$ can then be written as,

$$\rho(\mathbf{x}, t) = \sum_{i=1}^{N} \delta(\mathbf{x}_i(t) - \mathbf{x}(t)). \tag{S32}$$

Consider an arbitrary function $f(\mathbf{x}_i(t))$ defined on the coordinate space of the system. Using the definition of $\rho_i(\mathbf{x}, t)$, we can write,

$$f(\mathbf{x}_i(t)) = \int_{\mathbb{R}^d} f(\mathbf{x})\rho_i(\mathbf{x}, t)d\mathbf{x}. \tag{S33}$$

Expanding $f(\mathbf{x}_i(t))$ using Eq. S13 and Ito's lemma, we get,

$$\begin{aligned}\frac{df(\mathbf{x}_i(t))}{dt} = &-\frac{1}{\gamma}\sum_j \partial_{x_i,\alpha}V(\mathbf{x}_i, \mathbf{x}_j)\,\partial_{x_i,\alpha}f(\mathbf{x}_i(t)) + \sum_j \sqrt{\Lambda(\mathbf{x}_i, \mathbf{x}_j)}_{\alpha\beta}\,\xi_{ji,\beta}(t)\partial_{x_i,\alpha}f(\mathbf{x}_i(t)) \\ &+ \frac{1}{2}\partial_{x_i,\alpha}\partial_{x_i,\gamma}f(\mathbf{x}_i(t))\sum_j \sqrt{\Lambda(\mathbf{x}_i, \mathbf{x}_j)}_{\alpha\beta}\sqrt{\Lambda(\mathbf{x}_i, \mathbf{x}_j)}_{\gamma\beta}.\end{aligned} \tag{S34}$$

Using Eq. S33, the fact that $\sqrt{\Lambda(\mathbf{x}_i, \mathbf{x}_j)}_{\alpha\beta}\sqrt{\Lambda(\mathbf{x}_i, \mathbf{x}_j)}_{\gamma\beta} = \Lambda(\mathbf{x}_i, \mathbf{x}_j)_{\alpha\gamma}$, $\partial_{x_i,\alpha}f(\mathbf{x}_i) = \int \rho_i(\mathbf{x})\partial_{x,\alpha}f(\mathbf{x})d\mathbf{x}$, and that $\partial_{x_i,\alpha}\partial_{x_i,\gamma}f(\mathbf{x}_i) = \int \rho_i(\mathbf{x})\partial_{x,\alpha}\partial_{x,\gamma}f(\mathbf{x})d\mathbf{x}$, we get,

$$\begin{aligned}\frac{df(\mathbf{x}_i(t))}{dt} = \int d\mathbf{x}\,\rho_i(\mathbf{x}, t)\Bigg[&-\frac{1}{\gamma}\sum_j \partial_{x,\alpha}V(\mathbf{x}, \mathbf{x}_j)\,\partial_{x,\alpha}f(\mathbf{x}_i(t)) + \sum_j \sqrt{\Lambda(\mathbf{x}, \mathbf{x}_j)}_{\alpha\beta}\,\xi_{ji,\beta}(t)\,\partial_{x,\alpha}f(\mathbf{x}(t)) \\ &+ \frac{1}{2}\partial_{x,\alpha}\partial_{x,\gamma}f(\mathbf{x}(t))\sum_j \Lambda(\mathbf{x}, \mathbf{x}_j)_{\alpha\gamma}\Bigg].\end{aligned} \tag{S35}$$

Using integration by parts on Eq. S35 and assuming $f(|\mathbf{x}| \to \infty) = 0$, we get,

$$\frac{df(\mathbf{x}_i(t))}{dt} = \int d\mathbf{x}\, f(\mathbf{x}) \left[\frac{1}{\gamma} \partial_\alpha \left(\rho_i(\mathbf{x}) \sum_j \partial_\alpha V(\mathbf{x}, \mathbf{x}_j) \right) - \partial_\alpha \left(\rho_i(\mathbf{x}) \sum_j \sqrt{\Lambda(\mathbf{x}, \mathbf{x}_j)}_{\alpha\beta}\, \xi_{ji,\beta}(t) \right) \right.$$
$$\left. + \frac{1}{2} \partial_\alpha \partial_\gamma \left(\rho_i(\mathbf{x}) \sum_j \Lambda(\mathbf{x}, \mathbf{x}_j)_{\alpha\gamma} \right) \right], \tag{S36}$$

where $\partial_\alpha = \partial_{x,\alpha}$ hereafter. But, from Eq. S33, we also have,

$$\frac{df(\mathbf{x}^i(t))}{dt} = \int d\mathbf{x} \frac{\partial \rho^i(\mathbf{x}, t)}{\partial t} f(\mathbf{x}). \tag{S37}$$

Comparing Eqs. S36 and S37, we get,

$$\frac{\partial \rho_i(\mathbf{x}, t)}{\partial t} = \frac{1}{\gamma} \partial_\alpha \left(\rho_i(\mathbf{x}) \sum_j \partial_\alpha V(\mathbf{x}, \mathbf{x}_j) \right) - \partial_\alpha \left(\rho_i(\mathbf{x}) \sum_j \sqrt{\Lambda(\mathbf{x}, \mathbf{x}_j)}_{\alpha\beta}\, \xi_{ji,\beta}(t) \right) + \frac{1}{2} \partial_\alpha \partial_\gamma \left(\rho_i(\mathbf{x}) \sum_j \sqrt{\Lambda(\mathbf{x}, \mathbf{x}_j)}_{\alpha\gamma} \right). \tag{S38}$$

Summing Eq. S38 over $i = 1$ to N, we get,

$$\frac{\partial \rho(\mathbf{x}, t)}{\partial t} = \sum_i \frac{1}{\gamma} \partial_\alpha \left(\rho_i(\mathbf{x}) \sum_j \partial_\alpha V(\mathbf{x}, \mathbf{x}_j) \right) - \sum_i \partial_\alpha \left(\rho_i(\mathbf{x}) \sum_j \sqrt{\Lambda(\mathbf{x}, \mathbf{x}_j)}_{\alpha\beta}\, \xi_{ji,\beta}(t) \right) + \sum_i \frac{1}{2} \partial_\alpha \partial_\gamma \left(\rho_i(\mathbf{x}) \sum_j \Lambda(\mathbf{x}, \mathbf{x}_j)_{\alpha\gamma} \right). \tag{S39}$$

Consider the first term on the right hand side (RHS) of Eq. S39,

$$\begin{aligned}
\sum_i \frac{1}{\gamma} \partial_\alpha \left(\rho_i(\mathbf{x}) \sum_j \partial_\alpha V(\mathbf{x}, \mathbf{x}_j) \right) &= \frac{1}{\gamma} \partial_\alpha \left(\rho(\mathbf{x}) \sum_j \partial_\alpha V(\mathbf{x}, \mathbf{x}_j) \right) \\
&= \frac{1}{\gamma} \partial_\alpha \left(\rho(\mathbf{x}) \int \sum_j \partial_\alpha V(\mathbf{x}, \mathbf{y}) \delta(\mathbf{y} - \mathbf{x}_j) d\mathbf{y} \right) \\
&= \frac{1}{\gamma} \partial_\alpha \left(\rho(\mathbf{x}) \int \sum_j \partial_\alpha V(\mathbf{x}, \mathbf{y}) \rho_j(\mathbf{y}) d\mathbf{y} \right) \\
&= \frac{1}{\gamma} \partial_\alpha \left(\rho(\mathbf{x}) \int \rho(\mathbf{y}) \partial_\alpha V(\mathbf{x}, \mathbf{y}) d\mathbf{y} \right),
\end{aligned} \tag{S40}$$

where we have used Eq. S31, and the property of the delta function that $f(\mathbf{x}_i)\delta(\mathbf{x} - \mathbf{x}_i) = f(\mathbf{x})\delta(\mathbf{x} - \mathbf{x}_i)$. Consider the third term on the RHS of Eq. S39,

$$\begin{aligned}
\sum_i \frac{1}{2} \partial_\alpha \partial_\gamma \left(\rho_i(\mathbf{x}) \sum_j \Lambda(\mathbf{x}, \mathbf{x}_j)_{\alpha\gamma} \right) &= \frac{1}{2} \partial_\alpha \partial_\gamma \left(\rho(\mathbf{x}) \sum_j \Lambda(\mathbf{x}, \mathbf{x}_j)_{\alpha\gamma} \right) \\
&= \frac{1}{2} \partial_\alpha \partial_\gamma \left(\rho(\mathbf{x}) \int \sum_j \Lambda(\mathbf{x}, \mathbf{y})_{\alpha\gamma} \delta(\mathbf{y} - \mathbf{x}_j) d\mathbf{y} \right) \\
&= \frac{1}{2} \partial_\alpha \partial_\gamma \left(\rho(\mathbf{x}) \int \sum_j \Lambda(\mathbf{x}, \mathbf{y})_{\alpha\gamma} \rho_j(\mathbf{y}) d\mathbf{y} \right) \\
&= \frac{1}{2} \partial_\alpha \partial_\gamma \left(\rho(\mathbf{x}) \int \rho(\mathbf{y}) \Lambda(\mathbf{x}, \mathbf{y})_{\alpha\gamma} d\mathbf{y} \right).
\end{aligned} \tag{S41}$$

We next consider the second term on the RHS of Eq. S39,

$$\theta(\mathbf{x}, t) = -\sum_i \partial_\alpha \left(\rho_i(\mathbf{x}) \sum_j \sqrt{\Lambda(\mathbf{x}, \mathbf{x}_j)}_{\alpha\beta}\, \xi_{ji,\beta}(t) \right) = -\sum_i \sum_j \partial_\alpha \left(\rho_i(\mathbf{x}) \int \rho_j(\mathbf{y}) \sqrt{\Lambda(\mathbf{x}, \mathbf{y})}_{\alpha\beta} d\mathbf{y}\, \xi_{ji,\beta}(t) \right). \tag{S42}$$

Writing the correlation of $\theta(\mathbf{x},t)$, we get,

$$\begin{aligned}
\langle\theta(\mathbf{x},t)\theta(\mathbf{y},t')\rangle &= \sum_i\sum_j\sum_m\sum_n \partial_{x,\alpha}\partial_{y,\mu}\left[\rho_i(\mathbf{x})\,\rho_m(\mathbf{y})\int\rho_j(\mathbf{u})\sqrt{\Lambda(\mathbf{x},\mathbf{u})}_{\alpha\beta}d\mathbf{u}\int\rho_n(\mathbf{w})\sqrt{\Lambda(\mathbf{y},\mathbf{w})}_{\mu\nu}d\mathbf{w}\;\langle\xi_{ji,\beta}(t)\xi_{nm,\nu}(t')\rangle\right]\\
&= \delta(t-t')\sum_i\sum_j\partial_{x,\alpha}\partial_{y,\mu}\left[\rho_i(\mathbf{x})\,\rho_i(\mathbf{y})\int\rho_j(\mathbf{u})\sqrt{\Lambda(\mathbf{x},\mathbf{u})}_{\alpha\beta}d\mathbf{u}\int\rho_j(\mathbf{w})\sqrt{\Lambda(\mathbf{y},\mathbf{w})}_{\mu\beta}d\mathbf{w}\right]\\
&\quad + c\;\delta(t-t')\sum_i\sum_j\partial_{x,\alpha}\partial_{y,\mu}\left[\rho_i(\mathbf{x})\,\rho_j(\mathbf{y})\int\rho_j(\mathbf{u})\sqrt{\Lambda(\mathbf{x},\mathbf{u})}_{\alpha\beta}d\mathbf{u}\int\rho_i(\mathbf{w})\sqrt{\Lambda(\mathbf{y},\mathbf{w})}_{\mu\beta}d\mathbf{w}\right]\\
&= \delta(t-t')\partial_{x,\alpha}\partial_{y,\mu}\left[\delta(\mathbf{x}-\mathbf{y})\rho(\mathbf{x})\int\int\rho(\mathbf{u})\sqrt{\Lambda(\mathbf{x},\mathbf{u})}_{\alpha\beta}\sqrt{\Lambda(\mathbf{y},\mathbf{w})}_{\mu\beta}\,\delta(\mathbf{u}-\mathbf{w})d\mathbf{u}d\mathbf{w}\right]\\
&\quad + c\;\delta(t-t')\partial_{x,\alpha}\partial_{y,\mu}\left[\rho(\mathbf{x})\,\rho(\mathbf{y})\int\int\sqrt{\Lambda(\mathbf{x},\mathbf{u})}_{\alpha\beta}\sqrt{\Lambda(\mathbf{y},\mathbf{w})}_{\mu\beta}\,\delta(\mathbf{x}-\mathbf{w})\delta(\mathbf{y}-\mathbf{u})d\mathbf{u}d\mathbf{w}\right]\\
&= \delta(t-t')\partial_{x,\alpha}\partial_{y,\mu}\left[\delta(\mathbf{x}-\mathbf{y})\rho(\mathbf{x})\int\rho(\mathbf{u})\Lambda(\mathbf{x},\mathbf{u})_{\alpha\mu}d\mathbf{u}+c\,\rho(\mathbf{x})\rho(\mathbf{y})\Lambda(\mathbf{x},\mathbf{y})_{\alpha\mu}\right],
\end{aligned} \tag{S43}$$

where we have used the property of the delta function that $\delta(\mathbf{x}-\mathbf{x}_i)\delta(\mathbf{y}-\mathbf{x}_i)=\delta(\mathbf{x}-\mathbf{x}_i)\delta(\mathbf{x}-\mathbf{y})$. We aim to define a Gaussian noise field having a correlation function identical to Eq. S43 and is thus, statistically identical to $\theta(\mathbf{x},t)$. Consider a global noise $\chi(\mathbf{x},t)$ given as,

$$\chi(\mathbf{x},t)=\partial_\alpha\left(\sqrt{\rho(\mathbf{x})}\int\sqrt{\rho(\mathbf{u})}\sqrt{\Lambda(\mathbf{x},\mathbf{u})}_{\alpha\mu}\,\eta_\mu(\mathbf{x},\mathbf{u},t)\,d\mathbf{u}\right), \tag{S44}$$

where $\boldsymbol{\eta}(\mathbf{x},\mathbf{u},t)$ is a zero mean two-point Gaussian noise with correlation $\langle\eta_\alpha(\mathbf{x},\mathbf{u},t)\,\eta_\beta(\mathbf{y},\mathbf{w},t')\rangle=\delta_{\alpha\beta}\delta(t-t')\delta(\mathbf{x}-\mathbf{y})\delta(\mathbf{u}-\mathbf{w})+c\delta_{\alpha\beta}\delta(t-t')\delta(\mathbf{x}-\mathbf{w})\delta(\mathbf{u}-\mathbf{y})$. The correlation function of $\chi(\mathbf{x},t)$ is then given by,

$$\begin{aligned}
\langle\chi(\mathbf{x},t)\chi(\mathbf{y},t')\rangle &= \partial_{x,\alpha}\partial_{y,\mu}\left[\sqrt{\rho(\mathbf{x})\rho(\mathbf{y})}\int\int\sqrt{\rho(\mathbf{u})\rho(\mathbf{w})}\sqrt{\Lambda(\mathbf{x},\mathbf{u})}_{\alpha\beta}\sqrt{\Lambda(\mathbf{y},\mathbf{w})}_{\mu\nu}\,d\mathbf{u}d\mathbf{w}\,\langle\eta_\beta(\mathbf{x},\mathbf{u},t)\,\eta_\nu(\mathbf{y},\mathbf{w},t')\rangle\right]\\
&= \delta(t-t')\partial_{x,\alpha}\partial_{y,\mu}\left[\delta(\mathbf{x}-\mathbf{y})\sqrt{\rho(\mathbf{x})\rho(\mathbf{y})}\int\int\sqrt{\rho(\mathbf{u})\rho(\mathbf{w})}\sqrt{\Lambda(\mathbf{x},\mathbf{u})}_{\alpha\beta}\sqrt{\Lambda(\mathbf{y},\mathbf{w})}_{\mu\beta}\,\delta(\mathbf{u}-\mathbf{w})d\mathbf{u}d\mathbf{w}\right]\\
&\quad + c\delta(t-t')\partial_{x,\alpha}\partial_{y,\mu}\left[\rho(\mathbf{x})\,\rho(\mathbf{y})\int\int\sqrt{\Lambda(\mathbf{x},\mathbf{u})}_{\alpha\beta}\sqrt{\Lambda(\mathbf{y},\mathbf{w})}_{\mu\beta}\,\delta(\mathbf{x}-\mathbf{w})\delta(\mathbf{y}-\mathbf{u})d\mathbf{u}d\mathbf{w}\right]\\
&= \delta(t-t')\partial_{x,\alpha}\partial_{y,\mu}\left[\delta(\mathbf{x}-\mathbf{y})\rho(\mathbf{x})\int\rho(\mathbf{u})\Lambda(\mathbf{x},\mathbf{u})_{\alpha\mu}d\mathbf{u}+c\,\rho(\mathbf{x})\rho(\mathbf{y})\Lambda(\mathbf{x},\mathbf{y})_{\alpha\mu}\right].
\end{aligned} \tag{S45}$$

Eq. S45 is precisely the same as Eq. S43, showing that the noise terms $\theta(\mathbf{x},t)$ and $\chi(\mathbf{x},t)$ are statistically identical. Combining Eqs. S40, S41, and S44, we get the equation for the evolution of global density as,

$$\begin{aligned}
\frac{\partial\rho(\mathbf{x},t)}{\partial t} &= \frac{1}{\gamma}\,\partial_\alpha\left(\rho(\mathbf{x})\int\rho(\mathbf{y})\partial_\alpha V(\mathbf{x},\mathbf{y})d\mathbf{y}\right)+\frac{1}{2}\partial_\alpha\partial_\beta\left(\rho(\mathbf{x})\int\rho(\mathbf{y})\Lambda(\mathbf{x},\mathbf{y})_{\alpha\beta}d\mathbf{y}\right)\\
&\quad+\partial_\alpha\left(\sqrt{\rho(\mathbf{x})}\int\sqrt{\rho(\mathbf{y})}\sqrt{\Lambda(\mathbf{x},\mathbf{y})}_{\alpha\mu}\,\eta_\mu(\mathbf{x},\mathbf{y},t)\,d\mathbf{y}\right)\\
&= \underbrace{-\nabla\cdot\left[-\rho(\mathbf{x})\frac{\langle\nabla V(\mathbf{x},\mathbf{y})\rangle_{\rho(\mathbf{y})}}{\gamma}\right]}_{\text{drift term}}+\underbrace{\nabla\nabla:\left(\frac{1}{2}\langle\boldsymbol{\Lambda}(\mathbf{x},\mathbf{y})\rangle_{\rho(\mathbf{y})}\,\rho(\mathbf{x})\right)}_{\text{diffusion term}}\underbrace{-\nabla\cdot\left[-\sqrt{\rho(\mathbf{x})}\int\sqrt{\rho(\mathbf{y})}\sqrt{\boldsymbol{\Lambda}(\mathbf{x},\mathbf{y})}\cdot\boldsymbol{\eta}(\mathbf{x},\mathbf{y},t)d\mathbf{y}\right]}_{\text{noise term}},
\end{aligned} \tag{S46}$$

where : is the double dot product, and $\langle a\rangle_{\rho(\mathbf{y})}=\int a\rho(\mathbf{y})d\mathbf{y}$. Eq. S46 is Eq. 6 of the main text. Note that taking the ensemble average (over noise realizations) of Eq. S46, and using the mean-field approximation $\rho_2(\mathbf{x}_1,\mathbf{x}_2)=\rho_1(\mathbf{x}_1)\rho_1(\mathbf{x}_2)$ (see e.g., refs. [11–13]), we recover the density evolution equation derived previously using the Fokker-Planck method (Eq. S23, Sec. I.B.I).

Functional steady-state distribution. The functional Fokker-Planck equation for Eq. S46 is given by,

$$\frac{\partial P[\rho,t]}{\partial t} = \int d\mathbf{x}\frac{\delta}{\delta\rho(\mathbf{x})}\Bigg\{-\Bigg[\frac{1}{\gamma}\partial_{x,\alpha}\left(\rho(\mathbf{x})\int\rho(\mathbf{y})\partial_{x,\alpha}V(\mathbf{x},\mathbf{y})d\mathbf{y}\right)+\frac{1}{2}\partial_{x,\alpha}\partial_{x,\beta}\left(\rho(\mathbf{x})\int\rho(\mathbf{y})\Lambda_{\alpha\beta}(\mathbf{x},\mathbf{y})d\mathbf{y}\right)\Bigg]P$$
$$+\frac{1}{2}\int d\mathbf{y}\frac{\delta}{\delta\rho(\mathbf{y})}\left(\left[\partial_{x,\alpha}\partial_{y,\mu}\left(\delta(\mathbf{x}-\mathbf{y})\rho(\mathbf{x})\int\rho(\mathbf{u})\Lambda(\mathbf{x},\mathbf{u})_{\alpha\mu}d\mathbf{u}+c\,\rho(\mathbf{x})\rho(\mathbf{y})\Lambda(\mathbf{x},\mathbf{y})_{\alpha\mu}\right)\right]P\right)\Bigg\}, \tag{S47}$$

where $P[\rho,t]$ is the probability distribution functional of the density $\rho(\mathbf{x},t)$. Plugging the steady-state distribution functional P^s in Eq. S47 and noting that $\partial P^s/\partial t = 0$, we get,

$$\int d\mathbf{x}\frac{\delta}{\delta\rho(\mathbf{x})}\Bigg\{-\Bigg[\frac{1}{\gamma}\partial_{x,\alpha}\left(\rho(\mathbf{x})\int\rho(\mathbf{y})\partial_{x,\alpha}V(\mathbf{x},\mathbf{y})d\mathbf{y}\right)+\frac{1}{2}\partial_{x,\alpha}\partial_{x,\beta}\left(\rho(\mathbf{x})\int\rho(\mathbf{y})\Lambda_{\alpha\beta}(\mathbf{x},\mathbf{y})d\mathbf{y}\right)\Bigg]P^s$$
$$+\frac{1}{2}\int d\mathbf{y}\frac{\delta}{\delta\rho(\mathbf{y})}\left(\left[\partial_{x,\alpha}\partial_{y,\mu}\left(\delta(\mathbf{x}-\mathbf{y})\rho(\mathbf{x})\int\rho(\mathbf{u})\Lambda(\mathbf{x},\mathbf{u})_{\alpha\mu}d\mathbf{u}+c\,\rho(\mathbf{x})\rho(\mathbf{y})\Lambda(\mathbf{x},\mathbf{y})_{\alpha\mu}\right)\right]P^s\right)\Bigg\}=0. \tag{S48}$$

Eq. S48 is challenging to solve for general $\Lambda(\mathbf{x},\mathbf{y})_{\alpha\beta}$ and c, and no closed-form solution is known. However, substituting $\Lambda(\mathbf{x},\mathbf{y})_{\alpha\beta} = a\delta_{\alpha\beta}$, and $c = 0$ in Eq. S48, where a is some constant, we get,

$$\int d\mathbf{x}\frac{\delta}{\delta\rho(\mathbf{x})}\Bigg\{-\Bigg[\frac{1}{\gamma}\partial_{x,\alpha}\left(\rho(\mathbf{x})\int\rho(\mathbf{y})\partial_{x,\alpha}V(\mathbf{x},\mathbf{y})d\mathbf{y}\right)+\frac{aN}{2}\partial_{x,\alpha}\partial_{x,\alpha}\rho(\mathbf{x})\Bigg]P^s$$
$$+\frac{aN}{2}\int d\mathbf{y}\frac{\delta}{\delta\rho(\mathbf{y})}\left(\left[\partial_{x,\beta}\partial_{y,\beta}\left(\rho(\mathbf{x})\delta(\mathbf{x}-\mathbf{y})\right)\right]P^s\right)\Bigg\}=0, \tag{S49}$$

where $\int\rho(\mathbf{x})d\mathbf{x} = N$. Using integration by parts, and the fact that $\partial_{x,\alpha}f(\mathbf{x},\mathbf{y}) = -\partial_{y,\alpha}f(\mathbf{x},\mathbf{y})$ for any function f which depends only on $|\mathbf{x}-\mathbf{y}|$, we get,

$$\int d\mathbf{x}\frac{\delta}{\delta\rho(\mathbf{x})}\Bigg\{\Bigg[\frac{1}{\gamma}\partial_{x,\alpha}\left(\rho(\mathbf{x})\int\rho(\mathbf{y})\partial_{x,\alpha}V(\mathbf{x},\mathbf{y})d\mathbf{y}\right)+\frac{aN}{2}\partial_{x,\alpha}\partial_{x,\alpha}\rho(\mathbf{x})\Bigg]P^s+\frac{aN}{2}\partial_{x,\alpha}\left[\rho(\mathbf{x})\partial_{x,\alpha}\left(\frac{\delta P^s}{\delta\rho(\mathbf{x})}\right)\right]\Bigg\}=0 \tag{S50}$$

The solution of Eq. S50 is given by the Boltzmann distribution functional $P^s = P^B[\rho] \equiv (1/Z)e^{-(1/Na\gamma)F[\rho]}$, where Z is the normalization factor, and $F[\rho]$ is the free energy functional given by,

$$F[\rho] = Na\gamma\int\rho(\mathbf{x})\big(\ln\rho(\mathbf{x})-1\big)\,d\mathbf{x}+\iint\rho(\mathbf{x})\rho(\mathbf{y})V(\mathbf{x},\mathbf{y})d\mathbf{x}d\mathbf{y}. \tag{S51}$$

This can also be seen by the fact that substituting $\Lambda(\mathbf{x},\mathbf{y})_{\alpha\beta} = a\delta_{\alpha\beta}$, and $c = 0$ in Eq. S46 reduces it to a standard Dean's equation for a system of interacting Brownian particles at equilibrium, whose steady-state solution is known to be the Boltzmann distribution functional [7, 11]. However, since $\Lambda(\mathbf{x},\mathbf{y})_{\alpha\beta} \neq a\delta_{\alpha\beta}$, and $c \neq 0$, in general, for random-organizing systems (Table S1), the Boltzmann distribution functional with a time-independent temperature (as given by $P^B[\rho]$) is not the steady-state solution for Eq. S46.

Linearization. To make further analytical progress, we linearize the density to first order around a spatiotemporally constant mean density $\bar{\rho}$ as $\rho(\mathbf{x},t) = \bar{\rho}+\delta\rho(\mathbf{x},t)$. We plug this into Eq. S46 and retain terms up to the first order for the deterministic (first two on RHS) terms of Eq. S46. Further, we only retain the zeroth-order terms in the expansion of the noise (third on RHS) term in Eq. S46—since all the other terms in the expansion of the noise become $\mathcal{O}(\delta\rho^2)$ when taking correlations [14–17]. Notice that while the zeroth order contribution vanishes for the for the deterministic (first two on RHS) terms, it is non-zero for the noise (third on RHS) term (Eq. S46). We can then write the evolution of density fluctuation $\delta\rho(\mathbf{x},t)$ as,

$$\frac{\partial\delta\rho(\mathbf{x},t)}{\partial t} = \frac{\bar{\rho}}{\gamma}\,\partial_\alpha\left(\int\delta\rho(\mathbf{y},t)\partial_\alpha V(\mathbf{x}-\mathbf{y})\,d\mathbf{y}\right)+\frac{\bar{\rho}}{2}\partial_\alpha\partial_\beta\left(\int\delta\rho(\mathbf{y},t)\Lambda(\mathbf{x}-\mathbf{y})_{\alpha\beta}\,d\mathbf{y}+\delta\rho(\mathbf{x},t)\int\Lambda(\mathbf{x}-\mathbf{y})_{\alpha\beta}\,d\mathbf{y}\right)$$
$$+\bar{\rho}\,\partial_\alpha\left(\int\sqrt{\Lambda(\mathbf{x}-\mathbf{y})}_{\alpha\mu}\,\eta_\mu(\mathbf{x},\mathbf{y},t)\,d\mathbf{y}\right). \tag{S52}$$

We now define the Fourier transform in space and time for any function $f(\mathbf{x},t)$ as $\hat{f}(\mathbf{k},\omega)=\int\int f(\mathbf{x},t)e^{-i\mathbf{k}\cdot\mathbf{x}}e^{-i\omega t}d\mathbf{x}dt$. Taking Fourier transform of Eq. S52, we get,

$$\begin{aligned} i\omega\hat{\delta\rho}(\mathbf{k},\omega) &= -\frac{\bar{\rho}}{\gamma}|\mathbf{k}|^2\hat{\delta\rho}(\mathbf{k},\omega)\hat{V}(\mathbf{k}) - \frac{\bar{\rho}}{2}\hat{\delta\rho}(\mathbf{k},\omega)k_\alpha k_\beta\left(\hat{\Lambda}(\mathbf{k})_{\alpha\beta}+A_{\alpha\beta}\right) \\ &+ \bar{\rho}ik_\alpha\int e^{-i\mathbf{k}\cdot\mathbf{x}}d\mathbf{x}\int e^{-i\omega t}dt\int\sqrt{\Lambda(\mathbf{x}-\mathbf{y})}_{\alpha\mu}\,\eta_\mu(\mathbf{x},\mathbf{y},t)\,d\mathbf{y}, \end{aligned} \tag{S53}$$

where $A_{\alpha\beta}=\int\Lambda(\mathbf{r})_{\alpha\beta}\,d\mathbf{r}$. Rearranging Eq. S53, we get,

$$\hat{\delta\rho}(\mathbf{k},\omega)=\frac{\bar{\rho}ik_\alpha\int e^{-i\mathbf{k}\cdot\mathbf{x}}d\mathbf{x}\int e^{-i\omega t}dt\int\sqrt{\Lambda(\mathbf{x}-\mathbf{y})}_{\alpha\mu}\,\eta_\mu(\mathbf{x},\mathbf{y},t)\,d\mathbf{y}}{i\omega+\bar{\rho}\left[\frac{1}{\gamma}|\mathbf{k}|^2\hat{V}(\mathbf{k})+\frac{1}{2}k_\alpha k_\beta\left(\hat{\Lambda}(\mathbf{k})_{\alpha\beta}+A_{\alpha\beta}\right)\right]}. \tag{S54}$$

Structure factor. Defining f^* as the complex conjugate of any function f and using Eq. S54, we can write the dynamic structure factor $S(\mathbf{k},\omega)$ as,

$$\begin{aligned} S(\mathbf{k},\omega) &= \frac{1}{2\pi TN}\left\langle\hat{\delta\rho}(\mathbf{k},\omega)\hat{\delta\rho}^*(\mathbf{k},\omega)\right\rangle \\ &= \frac{\bar{\rho}^2k_\alpha k_\beta\left\langle\int e^{-i\mathbf{k}\cdot\mathbf{x}}d\mathbf{x}\int e^{-i\omega t}dt\int\sqrt{\Lambda(\mathbf{x}-\mathbf{y})}_{\alpha\mu}\,\eta_\mu(\mathbf{x},\mathbf{y},t)\,d\mathbf{y}\int e^{i\mathbf{k}\cdot\mathbf{x}'}d\mathbf{x}'\int e^{i\omega t'}dt'\int\sqrt{\Lambda(\mathbf{x}'-\mathbf{y}')}_{\beta\delta}\,\eta_\delta(\mathbf{x}',\mathbf{y}',t')\,d\mathbf{y}'\right\rangle}{2\pi TN\left(\omega^2+\bar{\rho}^2\left[\frac{1}{\gamma}|\mathbf{k}|^2\hat{V}(\mathbf{k})+\frac{1}{2}k_\alpha k_\beta\left(\hat{\Lambda}(\mathbf{k})_{\alpha\beta}+A_{\alpha\beta}\right)\right]^2\right)} \\ &= \frac{\bar{\rho}^2k_\alpha k_\beta\int\int\int\int\int\int e^{-i\mathbf{k}\cdot(\mathbf{x}-\mathbf{x}')}e^{-i\omega(t-t')}\sqrt{\Lambda(\mathbf{x}-\mathbf{y})}_{\alpha\mu}\sqrt{\Lambda(\mathbf{x}'-\mathbf{y}')}_{\beta\delta}\left\langle\eta_\mu(\mathbf{x},\mathbf{y},t)\eta_\delta(\mathbf{x}',\mathbf{y}',t')\right\rangle d\mathbf{x}d\mathbf{y}d\mathbf{x}'d\mathbf{y}'dtdt'}{2\pi TN\left(\omega^2+\bar{\rho}^2\left[\frac{1}{\gamma}|\mathbf{k}|^2\hat{V}(\mathbf{k})+\frac{1}{2}k_\alpha k_\beta\left(\hat{\Lambda}(\mathbf{k})_{\alpha\beta}+A_{\alpha\beta}\right)\right]^2\right)} \\ &= \frac{\bar{\rho}^2k_\alpha k_\beta\int\int\int\int\int\int e^{-i\mathbf{k}\cdot(\mathbf{x}-\mathbf{x}')}e^{-i\omega(t-t')}\sqrt{\Lambda(\mathbf{x}-\mathbf{y})}_{\alpha\mu}\sqrt{\Lambda(\mathbf{x}'-\mathbf{y}')}_{\beta\delta}\delta_{\mu\delta}\delta(t-t')\delta(\mathbf{x}-\mathbf{x}')\delta(\mathbf{y}-\mathbf{y}')d\mathbf{x}d\mathbf{y}d\mathbf{x}'d\mathbf{y}'dtdt'}{2\pi TN\left(\omega^2+\bar{\rho}^2\left[\frac{1}{\gamma}|\mathbf{k}|^2\hat{V}(\mathbf{k})+\frac{1}{2}k_\alpha k_\beta\left(\hat{\Lambda}(\mathbf{k})_{\alpha\beta}+A_{\alpha\beta}\right)\right]^2\right)} \\ &+ \frac{\bar{\rho}^2k_\alpha k_\beta c\int\int\int\int\int\int e^{-i\mathbf{k}\cdot(\mathbf{x}-\mathbf{x}')}e^{-i\omega(t-t')}\sqrt{\Lambda(\mathbf{x}-\mathbf{y})}_{\alpha\mu}\sqrt{\Lambda(\mathbf{x}'-\mathbf{y}')}_{\beta\delta}\delta_{\mu\delta}\delta(t-t')\delta(\mathbf{x}-\mathbf{y}')\delta(\mathbf{x}'-\mathbf{y})d\mathbf{x}d\mathbf{y}d\mathbf{x}'d\mathbf{y}'dtdt'}{2\pi TN\left(\omega^2+\bar{\rho}^2\left[\frac{1}{\gamma}|\mathbf{k}|^2\hat{V}(\mathbf{k})+\frac{1}{2}k_\alpha k_\beta\left(\hat{\Lambda}(\mathbf{k})_{\alpha\beta}+A_{\alpha\beta}\right)\right]^2\right)} \\ &= \frac{\bar{\rho}k_\alpha k_\beta\left(A_{\alpha\beta}+c\hat{\Lambda}(\mathbf{k})_{\alpha\beta}\right)}{2\pi\left(\omega^2+\bar{\rho}^2\left[\frac{1}{\gamma}|\mathbf{k}|^2\hat{V}(\mathbf{k})+\frac{1}{2}k_\alpha k_\beta\left(\hat{\Lambda}(\mathbf{k})_{\alpha\beta}+A_{\alpha\beta}\right)\right]^2\right)}, \end{aligned} \tag{S55}$$

where $\int dt=T$ is the total observation time, $N=\int\rho(\mathbf{x},t)\,d\mathbf{x}$ is the total number of particles, and we have used the fact that $\bar{\rho}=N/\int d\mathbf{x}$. We now write the static structure factor $S(\mathbf{k})$ as,

$$S(\mathbf{k})=\int S(\mathbf{k},\omega)\,d\omega=\frac{k_\alpha k_\beta\left(A_{\alpha\beta}+c\hat{\Lambda}(\mathbf{k})_{\alpha\beta}\right)}{2\left[\frac{1}{\gamma}|\mathbf{k}|^2\hat{V}(\mathbf{k})+\frac{1}{2}k_\alpha k_\beta\left(\hat{\Lambda}(\mathbf{k})_{\alpha\beta}+A_{\alpha\beta}\right)\right]}. \tag{S56}$$

Eq. S56 shows that $S(\mathbf{k})$ depends on the specific functional forms of $A_{\alpha\beta}=\int\Lambda(\mathbf{r})_{\alpha\beta}\,d\mathbf{r}$, $\hat{\Lambda}(\mathbf{k})_{\alpha\beta}$, and $\hat{V}(\mathbf{k})$. Since we are only interested in the long-range ($|\mathbf{k}|\to 0$) behavior of $S(\mathbf{k})$, we can approximate $\hat{V}(\mathbf{k})$ as,

$$\begin{aligned} \hat{V}(\mathbf{k}) &= \int V(\mathbf{r})e^{-i\mathbf{k}\cdot\mathbf{r}}d\mathbf{r}\approx\int V(\mathbf{r})\left(1-ik_\alpha r_\alpha-\frac{k_\alpha r_\alpha k_\beta r_\beta}{2}\right)d\mathbf{r}=\int V(r)d\mathbf{r}-\frac{k_\alpha k_\beta}{2}\int V(r)r^2n_\alpha n_\beta d\mathbf{r} \\ &= \underbrace{S_d\int V(r)r^{d-1}dr}_{V_1}-k^2\underbrace{\frac{S_d}{2d}\int V(r)r^{d+1}dr}_{V_2}=V_1-k^2V_2, \end{aligned} \tag{S57}$$

where $k=|\mathbf{k}|$, $r=|\mathbf{r}|$, $\mathbf{n}=\mathbf{r}/|\mathbf{r}|$ is the unit vector, V_1 and V_2 are constants, S_d is the surface area of a unit d-dimensional hypersphere. We have also used the fact that $V(\mathbf{r})$ only depends on r and is an even function of r—true for RO, BRO, and SGD (Eqs. S9, and S11).

Notice that $\mathbf{\Lambda}(\mathbf{r})$ for RO, BRO, and SGD can be generically written as $\Lambda_{\alpha\beta}(\mathbf{r}) = f_1(r)\delta_{\alpha\beta} + f_2(r)n_\alpha n_\beta$, where $\mathbf{n} = \mathbf{r}/|\mathbf{r}|$ is the unit vector (Eqs. S6, S8, and S12). Specifically, (i) for RO, $f_1(r) = \mathbf{1}_{(0,2R)}(r_{ij})\ \epsilon^2/3d\tau$, and $f_2(r) = 0$, (ii) for BRO, $f_1(r) = 0$, and $f_2(r) = \mathbf{1}_{(0,2R)}(r_{ij})\ \epsilon^2/12\tau$, and (iii) for SGD, $f_1(r) = 0$, and $f_2(r) = \mathbf{1}_{(0,2R)}(r_{ij})\ [E^2\alpha^2 b_f(1-b_f)/4R^2\tau](1-r/2R)^{2p-2}$. Then,

$$A_{\alpha\beta} = \int \Lambda(\mathbf{r})_{\alpha\beta} d\mathbf{r} = \int\int \left[f_1(r)\delta_{\alpha\beta} + f_2(r)n_\alpha n_\beta\right] r^{d-1} dS dr = \delta_{\alpha\beta} \underbrace{S_d \int \left(f_1(r) + \frac{f_2(r)}{d}\right) r^{d-1} dr}_{A_1} = A_1\delta_{\alpha\beta}, \quad \text{(S58)}$$

where A_1 is a constant. Since we are only interested in the long-range ($|\mathbf{k}| \to 0$) behavior of $S(\mathbf{k})$, we approximate $\hat{\Lambda}(\mathbf{k})_{\alpha\beta}$ as,

$$\begin{aligned}
\hat{\Lambda}(\mathbf{k})_{\alpha\beta} &= \int \Lambda(\mathbf{r})_{\alpha\beta} e^{-i\mathbf{k}\cdot\mathbf{r}} d\mathbf{r} \approx \int \Lambda(\mathbf{r})_{\alpha\beta}\left(1 - ik_\gamma r_\gamma - \frac{k_\gamma r_\gamma k_\delta r_\delta}{2}\right) d\mathbf{r} = A_{\alpha\beta} - \frac{k_\gamma k_\delta}{2}\int \Lambda(\mathbf{r})_{\alpha\beta} r^2 n_\gamma n_\delta d\mathbf{r} \\
&= A_1\delta_{\alpha\beta} - \frac{k_\gamma k_\delta}{2}\int\int \left[f_1(r)\delta_{\alpha\beta} + f_2(r)n_\alpha n_\beta\right] r^{d+1} n_\gamma n_\delta dS dr \\
&= A_1\delta_{\alpha\beta} - k^2\delta_{\alpha\beta} \underbrace{\frac{S_d}{2d}\int f_1(r) r^{d+1} dr}_{\lambda_1} - \left(k^2\delta_{\alpha\beta} + 2k_\alpha k_\beta\right) \underbrace{\frac{S_d}{2d(d+2)}\int f_2(r) r^{d+1} dr}_{\lambda_2} \\
&= A_1\delta_{\alpha\beta} - \lambda_1 k^2\delta_{\alpha\beta} - \lambda_2\left(k^2\delta_{\alpha\beta} + 2k_\alpha k_\beta\right), \qquad \text{(S59)}
\end{aligned}$$

where λ_1 and λ_2 are constants, and we have used the fact that $\Lambda(\mathbf{r})_{\alpha\beta}$ only depends on r and is an even function of r—true for RO, BRO, and SGD (Eqs. S6, S8, and S12). Finally, we combine Eqs. S57, S58, and S59 with Eq. S56 to get,

$$\begin{aligned}
S(\mathbf{k}) &= \frac{k_\alpha k_\beta\left(A_1\delta_{\alpha\beta} + c\left[A_1\delta_{\alpha\beta} - \lambda_1 k^2\delta_{\alpha\beta} - \lambda_2\left(k^2\delta_{\alpha\beta} + 2k_\alpha k_\beta\right)\right]\right)}{2\left[\frac{1}{\gamma}k^2(V_1 - k^2V_2) + \frac{1}{2}k_\alpha k_\beta\left(A_1\delta_{\alpha\beta} - \lambda_1 k^2\delta_{\alpha\beta} - \lambda_2\left(k^2\delta_{\alpha\beta} + 2k_\alpha k_\beta\right) + A_1\delta_{\alpha\beta}\right)\right]} \\
&= \frac{\left[(1+c)A_1 - c(\lambda_1 + 3\lambda_2)k^2\right]}{2\left[\frac{1}{\gamma}\left(V_1 - V_2k^2\right) + \frac{1}{2}\left(2A_1 - (\lambda_1 + 3\lambda_2)k^2\right)\right]} \\
&= \frac{1}{2}\left[(1+c)A_1 - c(\lambda_1 + 3\lambda_2)k^2\right]\left[\left(\frac{V_1}{\gamma} + A_1\right) - \left(\frac{V_2}{\gamma} + \frac{(\lambda_1 + 3\lambda_2)}{2}\right)k^2\right]^{-1} \\
&= \frac{1}{2\left(\frac{V_1}{\gamma A_1} + 1\right)}\left[(1+c) - \frac{c(\lambda_1 + 3\lambda_2)}{A_1}k^2\right]\left[1 + \left(\frac{\frac{V_2}{\gamma} + \frac{(\lambda_1 + 3\lambda_2)}{2}}{\frac{V_1}{\gamma} + A_1}\right)k^2 + \mathcal{O}(k^4)\right] \\
&= \frac{1}{2\left(\frac{V_1}{\gamma A_1} + 1\right)}\left[(1+c) + \left(\frac{\frac{V_2}{\gamma} + \frac{(\lambda_1 + 3\lambda_2)}{2}}{\frac{V_1}{\gamma} + A_1}\right)\left(1 + \left(1 - \frac{\frac{V_1}{\gamma A_1} + 1}{\frac{V_2}{\gamma(\lambda_1 + 3\lambda_2)} + \frac{1}{2}}\right)c\right)k^2 + \mathcal{O}(k^4)\right]. \qquad \text{(S60)}
\end{aligned}$$

We normalize S by the value of S for uncorrelated noise ($c = 0$) when $k \to 0$ to get $\tilde{S}(k) = S(k)/S_0$, where

$$S_0 = \frac{1}{2\left(\frac{V_1}{\gamma A_1} + 1\right)}. \qquad \text{(S61)}$$

We further normalize k by the value of k for which $\tilde{S}(c = -1) = \lim_{k\to 0}\tilde{S}(c = 0) = 1.0$ to get $\tilde{k} = k/k_0$, where

$$k_0 = \sqrt{\frac{A_1}{\lambda_1 + 3\lambda_2}}. \qquad \text{(S62)}$$

We then get,

$$\tilde{S}(\tilde{k}) = (1+c) + \left[\underbrace{\frac{\frac{V_2}{\gamma(\lambda_1 + 3\lambda_2)} + \frac{1}{2}}{\frac{V_1}{\gamma A_1} + 1}}_{M}(1+c) - c\right]\tilde{k}^2 + \mathcal{O}(\tilde{k}^4) = (1+c) + [M(1+c) - c]\tilde{k}^2 + \mathcal{O}(\tilde{k}^4), \qquad \text{(S63)}$$

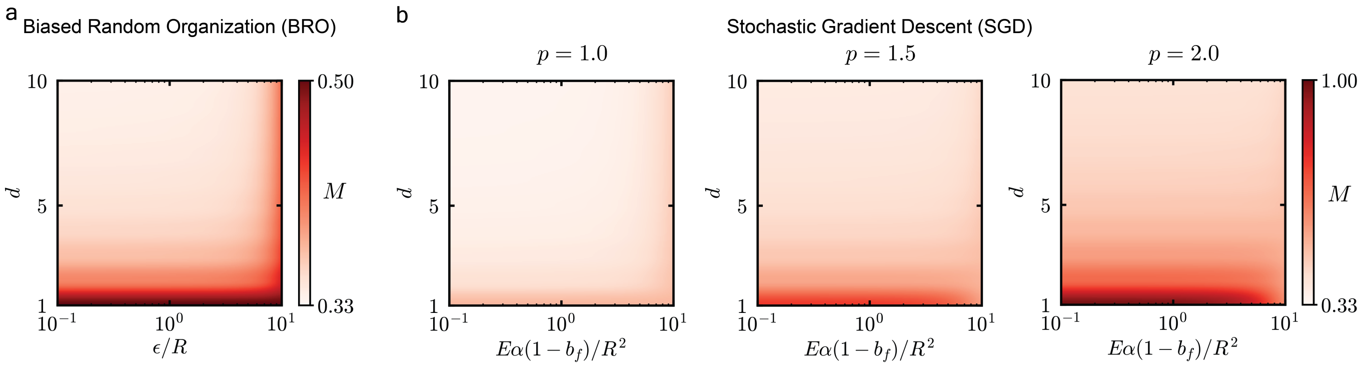

FIG. S1. (a) M (Eq. S66) for Biased Random Organization (BRO) as a function of spatial dimension d and ϵ/R, where R is the particle radius and ϵ is the kick magnitude. (b) M (Eq. S68) for Stochastic Gradient Descent (SGD) as a function of spatial dimension d and $E\alpha(1-b_f)/R^2$ for varying stiffness p of potential (Eq. S11). R is the particle radius, E is the characteristic energy scale, α is the learning rate, and b_f is the batch fraction.

where M is a system-specific constant. Neglecting the $\mathcal{O}(\tilde{k}^4)$ term, Eq. S63 yields Eq. 10 of the main text.

We now calculate M for RO, BRO, and SGD. For RO, $V_1^{\mathrm{RO}} = 0$ and $V_2^{\mathrm{RO}} = 0$. Using the definition of M, we then get,

$$M^{\mathrm{RO}} = \frac{1}{2}. \tag{S64}$$

For BRO,

$$\begin{aligned}
\gamma^{\mathrm{BRO}} &= \frac{\mathcal{E}\tau}{\epsilon R},\\
V_1^{\mathrm{BRO}} &= \frac{\mathcal{E}S_d(2R)^d}{d(d+1)},\\
V_2^{\mathrm{BRO}} &= \frac{\mathcal{E}S_d(2R)^{d+2}}{2d(d+2)(d+3)},\\
A_1^{\mathrm{BRO}} &= \frac{S_d\epsilon^2(2R)^d}{12\tau d^2},\\
\lambda_1^{\mathrm{BRO}} &= 0,\\
\lambda_2^{\mathrm{BRO}} &= \frac{S_d(2R)^{d+2},\epsilon^2}{24d(d+2)^2\tau}.
\end{aligned} \tag{S65}$$

Using the definition of M, we then get,

$$M^{\mathrm{BRO}} = \frac{(d+1)[8(d+2)+(d+3)\frac{\epsilon}{R}]}{2(d+3)[12d+(d+1)\frac{\epsilon}{R}]}. \tag{S66}$$

Fig. S1 shows that $M^{\mathrm{BRO}} \in (\frac{1}{3}, \frac{1}{2})$ for arbitrary spatial dimension d and ϵ/R, where R is the particle radius and ϵ is

the kick magnitude. This is also evident by taking the limits $\lim_{\epsilon/R\to 0}$ and $\lim_{\epsilon/R\to\infty}$ in Eq. S66. Similarly, for SGD,

$$\begin{aligned}
\gamma^{\rm SGD} &= \frac{\tau}{\alpha b_f},\\
V_1^{\rm SGD} &= \frac{\mathcal{E} S_d (2R)^d \Gamma(p+1)\Gamma(d)}{p\Gamma(p+d+1)},\\
V_2^{\rm SGD} &= \frac{\mathcal{E} S_d (2R)^{d+2} \Gamma(p+1)\Gamma(d+2)}{2dp\;\Gamma(p+d+3)},\\
A_1^{\rm SGD} &= \frac{S_d \mathcal{E}^2 \alpha^2 b_f (1-b_f)(2R)^d \Gamma(2p-1)\Gamma(d)}{4R^2 \tau d\;\Gamma(2p+d-1)},\\
\lambda_1^{\rm SGD} &= 0,\\
\lambda_2^{\rm SGD} &= \frac{S_d \mathcal{E}^2 \alpha^2 b_f (1-b_f)(2R)^{d+2} \Gamma(2p-1)\Gamma(d+2)}{8d(d+2)R^2\tau\;\Gamma(2p+d+1)},
\end{aligned} \tag{S67}$$

where Γ is the Gamma function. Using the definition of M, we then get,

$$M^{\rm SGD} = \frac{\frac{\mathcal{E}\alpha(1-b_f)}{R^2} 3p\Gamma(2p-1)\Gamma(p+d+3) + 8(d+2)\Gamma(p+1)\Gamma(2p+d+1)}{6(p+d+2)(p+d+1)\left[\frac{\mathcal{E}\alpha(1-b_f)}{R^2} p\Gamma(2p-1)\Gamma(p+d+1) + 4d\Gamma(p+1)\Gamma(2p+d-1)\right]}. \tag{S68}$$

Fig. S1 shows that for a linear potential ($p = 1$ in Eq. S11), $M^{\rm SGD} \in (\frac{1}{3}, \frac{1}{2})$ for arbitrary spatial dimension d and $E\alpha(1-b_f)/R^2$, where R is the particle radius, E is the characteristic energy scale, α is the learning rate, and b_f is the batch fraction. In general, for any non-linear potential ($p > 1$ in Eq. S11), $M^{\rm SGD} \in (\frac{1}{3}, 4)$ for arbitrary spatial dimension d and $E\alpha(1-b_f)/R^2$ (Fig. S1). This is also evident by taking the limits $\lim_{E\alpha(1-b_f)/R^2\to 0}$ and $\lim_{E\alpha(1-b_f)/R^2\to\infty}$ in Eq. S68.

C. Relationship between emergent structure and flatness of the energy landscape in SGD

Given a particle configuration ($\mathbf{X} \equiv \{\mathbf{x}_1, \mathbf{x}_2, ..., \mathbf{x}_N\}$) having energy $E(\mathbf{X}) = \sum_i \sum_{j\geq i} V_{ij}$, we aim to relate the change in energy $\Delta E(\mathbf{X}) = \langle E(\mathbf{X}+\Delta\mathbf{X}) - E(\mathbf{X})\rangle$ under a small Gaussian perturbation $\Delta\mathbf{X}$ to the structure factor $S(\mathbf{k})$ of the unperturbed configuration $\mathbf{X}$. $\Delta E(\mathbf{X})$ is a measure of the flatness of the energy landscape since, given V_{ij} is second-order smooth, $\Delta E(\mathbf{X}) \propto \mathrm{Tr}(\mathbf{H}(\mathbf{X}))$, where $\mathbf{H}(\mathbf{X})$ is the Hessian matrix [2, 18].

Since our system is in steady state, we are interested in the average energy over an ensemble of configurations $\langle . \rangle_{\mathbf{X}}$. We can then write [4],

$$E = \langle E(\mathbf{X})\rangle_{\mathbf{X}} = \frac{\rho N}{2}\int V(\mathbf{r}) g(\mathbf{r}) d\mathbf{r}, \tag{S69}$$

where $g(\mathbf{r})$ is the pair correlation function, $V(\mathbf{r}) = V(|\mathbf{r}_j - \mathbf{r}_i|)$ is the pairwise potential, ρ is the number density, and N is the total number of particles. Using $g(\mathbf{r}) - 1 = (1/\rho(2\pi)^d)\int [S(\mathbf{k}) - 1] e^{i\mathbf{k}\cdot\mathbf{r}} d\mathbf{k}$, we get [19],

$$\begin{aligned}
E &= \frac{\rho N}{2}\int V(\mathbf{r})d\mathbf{r} + \frac{\rho N}{2}\int V(\mathbf{r})\left(g(\mathbf{r}) - 1\right)d\mathbf{r}\\
&= \frac{\rho N}{2}\int V(\mathbf{r})d\mathbf{r} + \frac{N}{2(2\pi)^d}\int \hat{V}(\mathbf{k})\left(S(\mathbf{k}) - 1\right)d\mathbf{k}\\
&= \frac{\rho N}{2}\int V(\mathbf{r})d\mathbf{r} - \frac{N}{2}V(\mathbf{r}=\mathbf{0}) + \frac{N}{2(2\pi)^d}\int \hat{V}(\mathbf{k})S(\mathbf{k})d\mathbf{k},
\end{aligned} \tag{S70}$$

where we have used Parseval's theorem, the fact that $V(\mathbf{r})$ is an even function of $\mathbf{r}$, and that for any function $f(\mathbf{r})$, $1/(2\pi)^d \int \hat{f}(\mathbf{k})d\mathbf{k} = f(\mathbf{r}=\mathbf{0})$.

We now aim to relate E to the Pearson correlation coefficient c between the pairwise noise. For short-range potentials, $\hat{V}(\mathbf{k})$ is typically dominated by the small-k region; hence we can approximate $S(\mathbf{k})$ in Eq. S70 by the small-k (long-range) behavior. To proceed, we combine $S(\mathbf{k})$ derived from the linearized fluctuating hydrodynamic

theory (Eq. S63) with Eq. S70 to get,

$$
\begin{aligned}
E(c) &= \frac{\rho N}{2}\int V(\mathbf{r})d\mathbf{r} - \frac{N}{2(2\pi)^d}\int \hat{V}(\mathbf{k})\left(1 - S_0 - cS_0 - \frac{MS_0}{k_0^2}k^2 - \frac{cMS_0}{k_0^2}k^2 + \frac{cS_0}{k_0^2}k^2\right)d\mathbf{k} \\
&= \underbrace{\left(\frac{\rho N}{2}\int V(\mathbf{r})d\mathbf{r} - \frac{N\sigma^2}{2(2\pi)^d}\int \hat{V}(\mathbf{k})\left(1 - S_0 - \frac{MS_0}{k_0^2}k^2\right)d\mathbf{k}\right)}_{E_1} - c\underbrace{\left(\frac{NS_0}{2(2\pi)^d}\int \hat{V}(\mathbf{k})\left[(1-M)\frac{k^2}{k_0^2} - 1\right]d\mathbf{k}\right)}_{E_2} \\
&= E_1 - c\,E_2,
\end{aligned} \tag{S71}
$$

where E_1 and E_2 are system-dependent constants independent of c. To remove the dependence on E_1 and E_2, we normalize E as,

$$
\tilde{E}(c) = \frac{E(c) - E(c=0)}{E(c=-1) - E(c=0)} = 1 - c. \tag{S72}
$$

We next aim to relate E to the batch fraction b_f and learning rate α for anti-correlated noise ($c = -1$). Note that $c = -1$ and $b_f = 1$ correspond to (noiseless) gradient descent. We combine $S(\mathbf{k})$ derived from the linearized fluctuating hydrodynamic theory (Eq. S63) with Eq. S70 to get,

$$
\begin{aligned}
E(\alpha, b_f) &= \frac{\rho N}{2}\int V(\mathbf{r})d\mathbf{r} - \frac{N}{2(2\pi)^d}\int \hat{V}(\mathbf{k})\left(1 - S_0 - cS_0 - \frac{MS_0}{k_0^2}k^2 - \frac{cMS_0}{k_0^2}k^2 + \frac{cS_0}{k_0^2}k^2\right)d\mathbf{k} \\
&= \left(\frac{\rho N}{2}\int V(\mathbf{r})d\mathbf{r} - \frac{N}{2(2\pi)^d}\int \hat{V}(\mathbf{k})d\mathbf{k}\right) + S_0\left(\frac{N}{2(2\pi)^d}\int \hat{V}(\mathbf{k})\left[1 + c + [M + (M-1)c]\frac{k^2}{k_0^2}\right]d\mathbf{k}\right) \\
&= \underbrace{\left(\frac{\rho N}{2}\int V(\mathbf{r})d\mathbf{r} - \frac{N}{2(2\pi)^d}\int \hat{V}(\mathbf{k})d\mathbf{k}\right)}_{E_3} + S_0\underbrace{\left(\frac{N}{2(2\pi)^d}\int \hat{V}(\mathbf{k})\frac{k^2}{k_0^2}d\mathbf{k}\right)}_{E_4} \\
&= E_3 + \underbrace{\frac{1}{2\left(1 + \frac{4R^2 d\,\Gamma(p+1)\Gamma(2p+d-1)}{E\alpha(1-b_f)p\Gamma(2p-1)\Gamma(p+d+1)}\right)}}_{g(\alpha, b_f)} E_4,
\end{aligned} \tag{S73}
$$

where we substitute $c = -1$ on the third step, use Eqs. S61 and the fact that k_0 is independent of α and b_f (Eqs. S62, and S67). E_3 and E_4 are constants independent of α and b_f, and $g(\alpha, b_f)$ depends on α and b_f. It is evident by taking $\lim_{E\alpha(1-b_f)/R^2 \to 0}$ and $\lim_{E\alpha(1-b_f)/R^2 \to \infty}$ in Eq. S73 that $g(\alpha, b_f) \in (0, \frac{1}{2})$ for any arbitrary p and d. It is also evident from Eq. S73 that $E(\alpha, b_f)$ decreases monotonically with b_f, and increases monotonically with α. For a fixed b_f, to remove the dependence on E_3 and E_4, we normalize $E(\alpha)$ as,

$$
\tilde{E}(\alpha) = \frac{E(\alpha) - E(\alpha_{\min})}{E(\alpha_{\max}) - E(\alpha_{\min})} = \frac{g(\alpha) - g(\alpha_{\min})}{g(\alpha_{\max}) - g(\alpha_{\min})}. \tag{S74}
$$

Similarly, for a fixed α, we normalize $E(b_f)$ as,

$$
\tilde{E}(b_f) = \frac{E(b_f) - E(b_{f\max})}{E(b_{f\min}) - E(b_{f\max})} = \frac{g(b_f) - g(b_{f\max})}{g(b_{f\min}) - g(b_{f\max})}. \tag{S75}
$$

We now perturb the configuration $\mathbf{X} \equiv \{\mathbf{x}_1, \mathbf{x}_2, ..., \mathbf{x}_N\}$ by adding an independent Gaussian noise $\mathbf{N}_i(\mathbf{0}, \sigma^2\mathbf{I})$ to the position of each particle to get $\mathbf{X} + \Delta\mathbf{X} \equiv \{\mathbf{x}_1 + \mathbf{N}_1, \mathbf{x}_2 + \mathbf{N}_2, ..., \mathbf{x}_N + \mathbf{N}_N\}$. The new structure factor $S'(\mathbf{k})$ of the system after the addition of independent noise to all particles is given by [20–22],

$$
\begin{aligned}
S'(\mathbf{k}) &= 1 + |F(\mathbf{k})|^2\,(S(\mathbf{k}) - 1) \\
&= 1 + e^{-\sigma^2|\mathbf{k}|^2}\,(S(\mathbf{k}) - 1) \\
&= 1 + (1 - \sigma^2k^2 + \mathcal{O}(k^4))\,(S(\mathbf{k}) - 1) \\
&= \sigma^2k^2 + \left(1 - \sigma^2k^2\right)S(\mathbf{k}),
\end{aligned} \tag{S76}
$$

where $F(\mathbf{k}) = e^{-(\sigma^2/2)|\mathbf{k}|^2}$ is the characteristic function of distribution of noise added to particle positions (here, Gaussian noise), and we have dropped terms of order higher than $\mathcal{O}(k^2)$. Using Eqs. S70 and S76, we can now write

ΔE as,

$$\begin{aligned}\Delta E &= \langle E(\mathbf{X}+\Delta\mathbf{X}) - E(\mathbf{X})\rangle_{\mathbf{X},\mathbf{N}} \\ &= \frac{N}{2(2\pi)^d}\int \hat{V}(\mathbf{k})\left[S'(\mathbf{k}) - S(\mathbf{k})\right] d\mathbf{k}, \\ &= \frac{N\sigma^2}{2(2\pi)^d}\int \hat{V}(\mathbf{k})k^2\left[1 - S(\mathbf{k})\right] d\mathbf{k}. \end{aligned} \tag{S77}$$

Given a system quantified by $S(\mathbf{k})$, Eq. S77 relates $S(\mathbf{k})$ to the energy change ΔE when the system is perturbed by a small amount.

We now aim to relate ΔE to the Pearson correlation coefficient c between the pairwise noise. To proceed, we combine $S(\mathbf{k})$ derived from the linearized fluctuating hydrodynamic theory (Eq. S63) with Eq. S77 to get,

$$\begin{aligned}\Delta E(c) &= \frac{N\sigma^2}{2(2\pi)^d}\int \hat{V}(\mathbf{k})k^2\left(1 - S_0 - cS_0 - \frac{MS_0}{k_0^2}k^2 - \frac{cMS_0}{k_0^2}k^2 + \frac{cS_0}{k_0^2}k^2\right) d\mathbf{k} \\ &= \underbrace{\left(\frac{N\sigma^2}{2(2\pi)^d}\int \hat{V}(\mathbf{k})k^2\left(1 - S_0 - \frac{MS_0}{k_0^2}k^2\right) d\mathbf{k}\right)}_{\Delta E_1} + c\underbrace{\left(\frac{N\sigma^2 S_0}{2(2\pi)^d}\int \hat{V}(\mathbf{k})k^2\left[(1-M)\frac{k^2}{k_0^2} - 1\right] d\mathbf{k}\right)}_{\Delta E_2} \\ &= \Delta E_1 + c\,\Delta E_2, \end{aligned} \tag{S78}$$

where ΔE_1 and ΔE_2 are system-dependent constants independent of c. To remove the dependence on ΔE_1 and ΔE_2, we normalize ΔE as,

$$\Delta\tilde{E}(c) = \frac{\Delta E(c) - \Delta E(c=-1)}{\Delta E(c=0) - \Delta E(c=-1)} = 1 + c. \tag{S79}$$

We next aim to relate ΔE to the batch fraction b_f and learning rate α for anti-correlated noise ($c = -1$). Note that $c = -1$ and $b_f = 1$ correspond to (noiseless) gradient descent. We combine $S(\mathbf{k})$ derived from the linearized fluctuating hydrodynamic theory (Eq. S63) with Eq. S77 to get,

$$\begin{aligned}\Delta E(\alpha, b_f) &= \frac{N\sigma^2}{2(2\pi)^d}\int \hat{V}(\mathbf{k})k^2\left(1 - S_0 - cS_0 - \frac{MS_0}{k_0^2}k^2 - \frac{cMS_0}{k_0^2}k^2 + \frac{cS_0}{k_0^2}k^2\right) d\mathbf{k} \\ &= \left(\frac{N\sigma^2}{2(2\pi)^d}\int \hat{V}(\mathbf{k})k^2 d\mathbf{k}\right) - S_0\left(\frac{N\sigma^2}{2(2\pi)^d}\int \hat{V}(\mathbf{k})k^2\left[1 + c + [M + (M-1)c]\frac{k^2}{k_0^2}\right] d\mathbf{k}\right) \\ &= \underbrace{\left(\frac{N\sigma^2}{2(2\pi)^d}\int \hat{V}(\mathbf{k})k^2 d\mathbf{k}\right)}_{\Delta E_3} - S_0\underbrace{\left(\frac{N\sigma^2}{2(2\pi)^d}\int \hat{V}(\mathbf{k})\frac{k^4}{k_0^2} d\mathbf{k}\right)}_{\Delta E_4} \\ &= \Delta E_3 - \underbrace{\frac{1}{2\left(1 + \frac{4R^2 d\,\Gamma(p+1)\Gamma(2p+d-1)}{E\alpha(1-b_f)p\Gamma(2p-1)\Gamma(p+d+1)}\right)}}_{g(\alpha, b_f)}\Delta E_4, \end{aligned} \tag{S80}$$

where we substitute $c = -1$ on the third step, use Eqs. S61 and the fact that k_0 is independent of α and b_f (Eqs. S62, and S67). ΔE_3 and ΔE_4 are constants independent of α and b_f, and $g(\alpha, b_f)$ depends on α and b_f. It is evident from Eq. S80 that $\Delta E(\alpha, b_f)$ increases monotonically with b_f, and decreases monotonically with α. For a fixed b_f, to remove the dependence on ΔE_3 and ΔE_4, we normalize $\Delta E(\alpha)$ as,

$$\Delta\tilde{E}(\alpha) = \frac{\Delta E(\alpha) - \Delta E(\alpha_{\max})}{\Delta E(\alpha_{\min}) - \Delta E(\alpha_{\max})} = \frac{g(\alpha) - g(\alpha_{\max})}{g(\alpha_{\min}) - g(\alpha_{\max})}. \tag{S81}$$

Similarly, for a fixed α, we normalize $\Delta E(b_f)$ as,

$$\Delta\tilde{E}(b_f) = \frac{\Delta E(b_f) - \Delta E(b_{f\,\min})}{\Delta E(b_{f\,\max}) - \Delta E(b_{f\,\min})} = \frac{g(b_f) - g(b_{f\,\min})}{g(b_{f\,\max}) - g(b_{f\,\min})}. \tag{S82}$$

D. Effect of thermal noise

How is the coarse-grained density evolution and consequently, the long-range structure quantified by $S(k)$ affected by the addition of thermal noise in random-organizing systems? We incorporate thermal noise, $\sqrt{2D^{\mathrm{th}}}\,\boldsymbol{\xi}_i^{\mathrm{th}}(t)$, into the generalized model (Eq. S13), where $\boldsymbol{\xi}_i^{\mathrm{th}}(t)$ is a Gaussian noise having mean $\langle \xi_{i,\alpha}^{\mathrm{th}}(t)\rangle = 0$ and covariance matrix $\left\langle \xi_{i,\alpha}^{\mathrm{th}}(t)\,\xi_{j,\beta}^{\mathrm{th}}(t')\right\rangle = \delta(t-t')\,\delta_{\alpha\beta}\,\delta_{ij}$, $D^{\mathrm{th}} = k_B T/\gamma$, k_B is the Boltzmann constant, and T is the temperature, to get,

$$\frac{d\mathbf{x}_i(t)}{dt} = \underbrace{-\frac{1}{\gamma}\sum_{j=1}^{N}\nabla_i V_{ji}}_{\text{deterministic term}} + \underbrace{\sum_{j=1}^{N}\sqrt{\boldsymbol{\Lambda}_{ji}}\cdot\boldsymbol{\xi}_{ji}}_{\text{athermal noise term}} + \underbrace{\sqrt{2D^{\mathrm{th}}}\,\boldsymbol{\xi}_i^{\mathrm{th}}}_{\text{thermal noise term}} . \tag{S83}$$

We assume that the noise term in random-organizing systems (athermal noise) $\boldsymbol{\xi}_{ji}$ and thermal noise $\boldsymbol{\xi}_i^{\mathrm{th}}$ are uncorrelated, i.e., $\left\langle \xi_{ij,\alpha}(t)\,\xi_{k,\beta}^{\mathrm{th}}(t')\right\rangle = 0_{\alpha\beta}$. Following the coarse-graining as described in Sec. I.B.2, we get the equation for the density evolution,

$$\begin{aligned}
\frac{\partial\rho(\mathbf{x},t)}{\partial t} &= \frac{1}{\gamma}\,\partial_\alpha\left(\rho(\mathbf{x})\int\rho(\mathbf{y})\partial_\alpha V(\mathbf{x},\mathbf{y})d\mathbf{y}\right) + \frac{1}{2}\partial_\alpha\partial_\beta\left(\rho(\mathbf{x})\int\rho(\mathbf{y})\Lambda(\mathbf{x},\mathbf{y})_{\alpha\beta}d\mathbf{y}\right) + D^{\mathrm{th}}\partial_\alpha\partial_\alpha\left(\rho(\mathbf{x})\right) \\
&\quad + \partial_\alpha\left(\sqrt{\rho(\mathbf{x})}\int\sqrt{\rho(\mathbf{y})}\sqrt{\Lambda(\mathbf{x},\mathbf{y})}_{\alpha\mu}\,\eta_\mu(\mathbf{x},\mathbf{y},t)\,d\mathbf{y}\right) + \sqrt{2D^{\mathrm{th}}}\,\partial_\alpha\left(\sqrt{\rho(\mathbf{x})}\,\eta_\alpha^{\mathrm{th}}(\mathbf{x},t)\right) \\
&= \underbrace{-\nabla\cdot\left[-\rho(\mathbf{x})\frac{\langle\nabla V(\mathbf{x},\mathbf{y})\rangle_{\rho(\mathbf{y})}}{\gamma}\right]}_{\text{drift term}} + \underbrace{\nabla\nabla:\left(\frac{1}{2}\left\langle\boldsymbol{\Lambda}(\mathbf{x},\mathbf{y})\right\rangle_{\rho(\mathbf{y})}\rho(\mathbf{x})\right)}_{\text{athermal diffusion term}} + \underbrace{D^{\mathrm{th}}\nabla^2\left(\rho(\mathbf{x})\right)}_{\text{thermal diffusion term}} \\
&\quad \underbrace{-\nabla\cdot\left[-\sqrt{\rho(\mathbf{x})}\int\sqrt{\rho(\mathbf{y})}\sqrt{\boldsymbol{\Lambda}(\mathbf{x},\mathbf{y})}\cdot\boldsymbol{\eta}(\mathbf{x},\mathbf{y},t)d\mathbf{y}\right]}_{\text{athermal noise term}} \underbrace{-\sqrt{2D^{\mathrm{th}}}\,\nabla\cdot\left[-\sqrt{\rho(\mathbf{x})}\,\boldsymbol{\eta}^{\mathrm{th}}(\mathbf{x},t)\right]}_{\text{thermal noise term}},
\end{aligned} \tag{S84}$$

where : is the double dot product, and $\langle a\rangle_{\rho(\mathbf{y})} = \int a\rho(\mathbf{y})d\mathbf{y}$. Comparing Eq. S46 and Eq. S84, it is evident that the addition of thermal noise leads to two new terms in the density evolution equation, namely, the thermal diffusion and noise term. Following the density linearization as described in Sec. I.B.2, we get the new normalized structure factor as,

$$\begin{aligned}
\tilde{S}(\tilde{k}) &= \underbrace{\frac{\frac{V_1}{\gamma A_1}+1}{\frac{V_1}{\gamma A_1}+\frac{D^{\mathrm{th}}}{\bar{\rho}A_1}+1}}_{M_1^{\mathrm{th}}}\left[1+c+\frac{2D^{\mathrm{th}}}{\bar{\rho}A_1}+\underbrace{\left(\frac{\frac{V_2}{\gamma(\lambda_1+3\lambda_2)}+\frac{1}{2}}{\frac{V_1}{\gamma A_1}+\frac{D^{\mathrm{th}}}{\bar{\rho}A_1}+1}\right)}_{M_2^{\mathrm{th}}}\left(1+\frac{2D^{\mathrm{th}}}{\bar{\rho}A_1}+\left[1-\frac{\frac{V_1}{\gamma A_1}+\frac{D^{\mathrm{th}}}{\bar{\rho}A_1}+1}{\frac{V_2}{\gamma(\lambda_1+3\lambda_2)}+\frac{1}{2}}\right]c\right)\tilde{k}^2\right] \\
&= M_1^{\mathrm{th}}\left[1+c+\frac{2D^{\mathrm{th}}}{\bar{\rho}A_1}+\left[M_2^{\mathrm{th}}\left(1+\frac{2D^{\mathrm{th}}}{\bar{\rho}A_1}+c\right)-c\right]\tilde{k}^2\right],
\end{aligned} \tag{S85}$$

where all symbols mean the same as described in Sec. I.B.2. Notice that substituting $D^{\mathrm{th}} = 0$ in Eq. S85 immediately reduces it to Eq. S63.

---

[1] Q. Li, C. Tai, and E. Weinan, in *International Conference on Machine Learning* (PMLR, 2017) pp. 2101–2110.
[2] G. Zhang and S. Martiniani, arXiv preprint arXiv:2411.11834 (2024).
[3] I. Sadrtdinov, I. Klimov, E. Lobacheva, and D. Vetrov, arXiv preprint arXiv:2505.23489 (2025).
[4] J.-P. Hansen and I. R. McDonald, *Theory of simple liquids: with applications to soft matter* (Academic press, 2013).
[5] P.-H. Chavanis, Physica A: Statistical Mechanics and its Applications **390**, 1546 (2011).
[6] H. Risken, The fokker-planck equation (1996).
[7] D. S. Dean, Journal of Physics A: Mathematical and General **29**, L613 (1996).
[8] E. Bertin, H. Chaté, F. Ginelli, S. Mishra, A. Peshkov, and S. Ramaswamy, New journal of physics **15**, 085032 (2013).
[9] A. P. Solon, M. E. Cates, and J. Tailleur, The European Physical Journal Special Topics **224**, 1231 (2015).

[10] A. Donev and E. Vanden-Eijnden, The Journal of chemical physics **140** (2014).
[11] P. Illien, arXiv preprint arXiv:2411.13467 (2024).
[12] M. Krüger, A. Solon, V. Démery, C. M. Rohwer, and D. S. Dean, The Journal of chemical physics **148** (2018).
[13] U. M. B. Marconi and P. Tarazona, The Journal of chemical physics **110**, 8032 (1999).
[14] D. S. Dean, B.-S. Lu, A. Maggs, and R. Podgornik, Physical Review Letters **116**, 240602 (2016).
[15] D. S. Dean and R. Podgornik, Physical Review E **89**, 032117 (2014).
[16] V. Démery, O. Bénichou, and H. Jacquin, New Journal of Physics **16**, 053032 (2014).
[17] A. Dinelli, J. O'Byrne, and J. Tailleur, Journal of Physics A: Mathematical and Theoretical **57**, 395002 (2024).
[18] S. Jastrzębski, Z. Kenton, D. Arpit, N. Ballas, A. Fischer, Y. Bengio, and A. Storkey, arXiv preprint arXiv:1711.04623 (2017).
[19] S. Torquato, G. Zhang, and F. H. Stillinger, Physical Review X **5**, 021020 (2015).
[20] A. Gabrielli, Physical Review E—Statistical, Nonlinear, and Soft Matter Physics **70**, 066131 (2004).
[21] J. Kim and S. Torquato, Physical Review B **97**, 054105 (2018).
[22] M. Casiulis, A. Shih, and S. Martiniani, Physical Review Letters **135**, 196101 (2025).

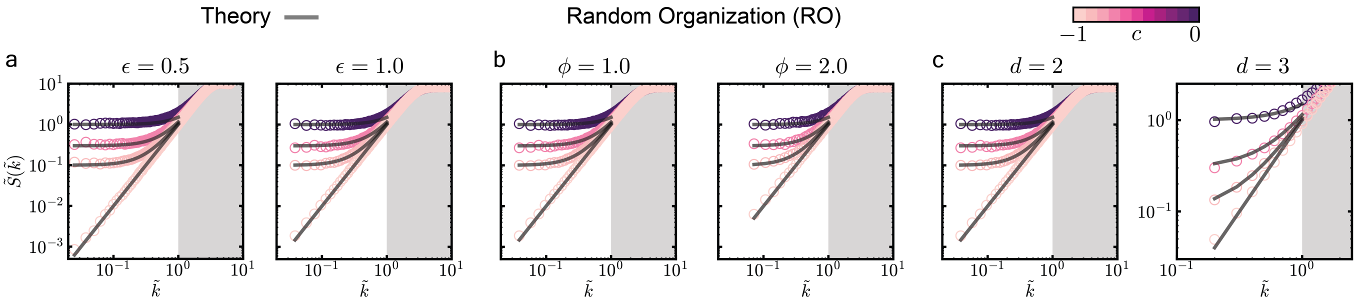

FIG. S2. Dependence of the structure factor on system parameters for Random Organization (RO). Normalized radially averaged structure factor $\tilde{S}(\tilde{k})$ versus normalized radial wave number $\tilde{k}$ for different kick magnitude ϵ (a), particle volume fraction ϕ (b), and spatial dimension d (c) in discrete-time particle simulations. Baseline parameters are set to $N = 318309$, $\epsilon = 1.0$, $\phi = 1.0$, $d = 2$, and each panel varies one parameter. For $d = 3$, we choose $N = 2546472$ to better explore large length scales. $\tilde{S} = S(k)/S_0(2\pi/L)$ where $S_0(2\pi/L)$ is the structure factor for $c = 0$ at $k = 2\pi/L$, and L is the side length of the simulation box. $\tilde{k} = k/k_0$ where k_0 is the value at which $\tilde{S}(k_0) = 1$ for anti-correlated noise ($c = -1$) of the same system. Solid black lines show predictions of Eq. S63 for different values of c, where M is substituted from Eq. S64. Gray shaded regions denote short-range behavior ($\tilde{k} > 1$).

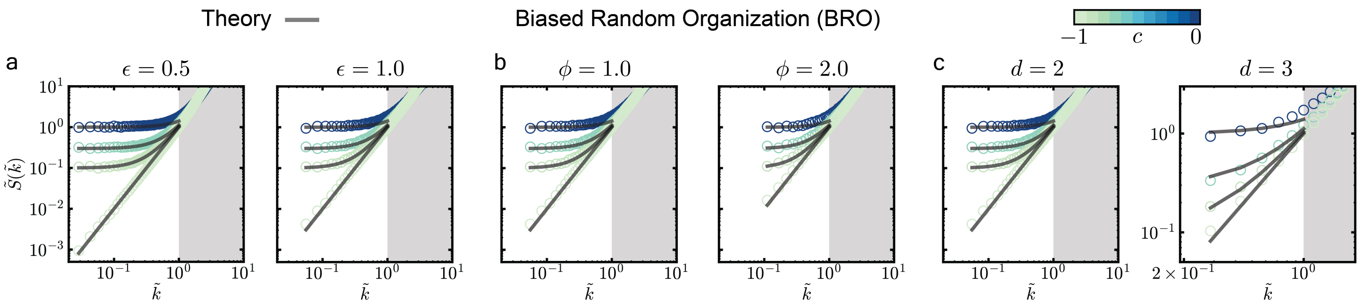

FIG. S3. Dependence of the structure factor on system parameters for Biased Random Organization (BRO). Normalized radially averaged structure factor $\tilde{S}(\tilde{k})$ versus normalized radial wave number $\tilde{k}$ for different kick magnitude ϵ (a), particle volume fraction ϕ (b), and spatial dimension d (c) in discrete-time particle simulations. Baseline parameters are set to $N = 318309$, $\epsilon = 1.0$, $\phi = 1.0$, $d = 2$, and each panel varies one parameter. For $d = 3$, we choose $N = 2546472$ to better explore large length scales. $\tilde{S} = S(k)/S_0(2\pi/L)$ where $S_0(2\pi/L)$ is the structure factor for $c = 0$ at $k = 2\pi/L$, and L is the side length of the simulation box. $\tilde{k} = k/k_0$ where k_0 is the value at which $\tilde{S}(k_0) = 1$ for anti-correlated noise ($c = -1$) of the same system. Solid black lines show predictions of Eq. S63 for different values of c, where M is substituted from Eq. S66. Gray shaded regions denote short-range behavior ($\tilde{k} > 1$).

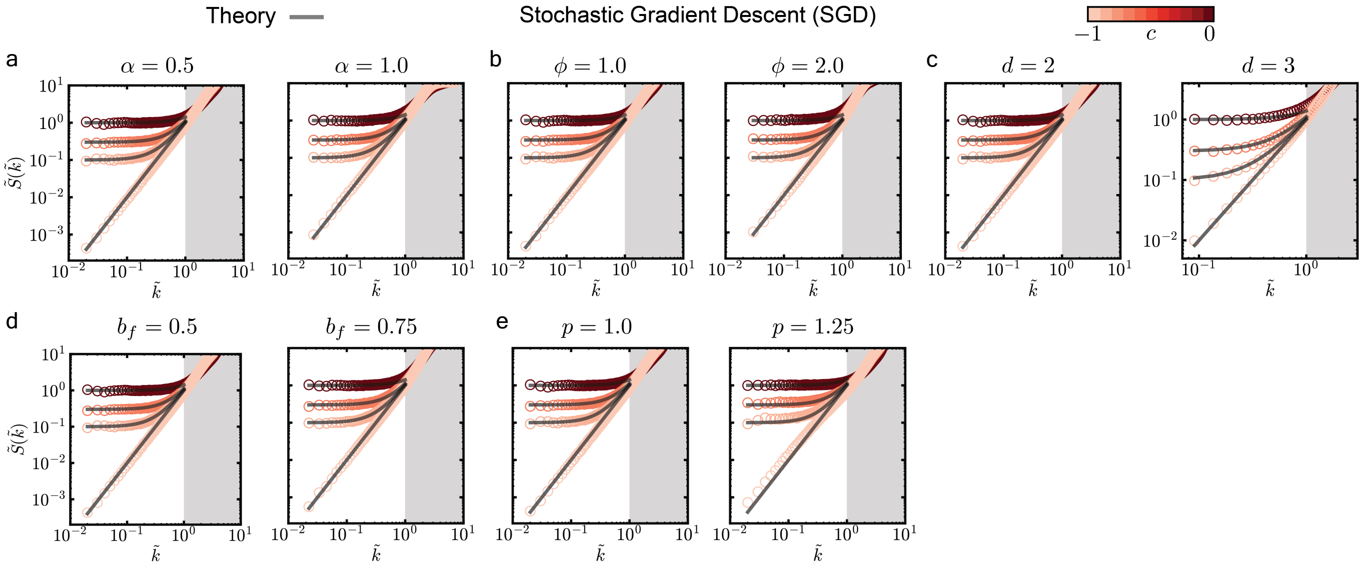

FIG. S4. Dependence of the structure factor on system parameters for Stochastic Gradient Descent (SGD). Normalized radially averaged structure factor $\tilde{S}(\tilde{k})$ versus normalized radial wave number $\tilde{k}$ for different learning rate α (a), particle volume fraction ϕ (b), spatial dimension d (c), batch fraction b_f (d), and stiffness of the potential p (Eq. S11) (e) in discrete-time particle simulations. Baseline parameters are set to $N = 318309$, $\alpha = 0.5$, $\phi = 1.0$, $d = 2$, $b_f = 0.5$, $p = 1.0$, and each panel varies one parameter. For $d = 3$, we choose $N = 2546472$ to better explore large length scales. $\tilde{S} = S(k)/S_0(2\pi/L)$ where $S_0(2\pi/L)$ is the structure factor for $c = 0$ at $k = 2\pi/L$, and L is the side length of the simulation box. $\tilde{k} = k/k_0$ where k_0 is the value at which $\tilde{S}(k_0) = 1$ for anti-correlated noise ($c = -1$) of the same system. Solid black lines show predictions of Eq. S63 for different values of c, where M is substituted from Eq. S68. Gray shaded regions denote short-range behavior ($\tilde{k} > 1$).

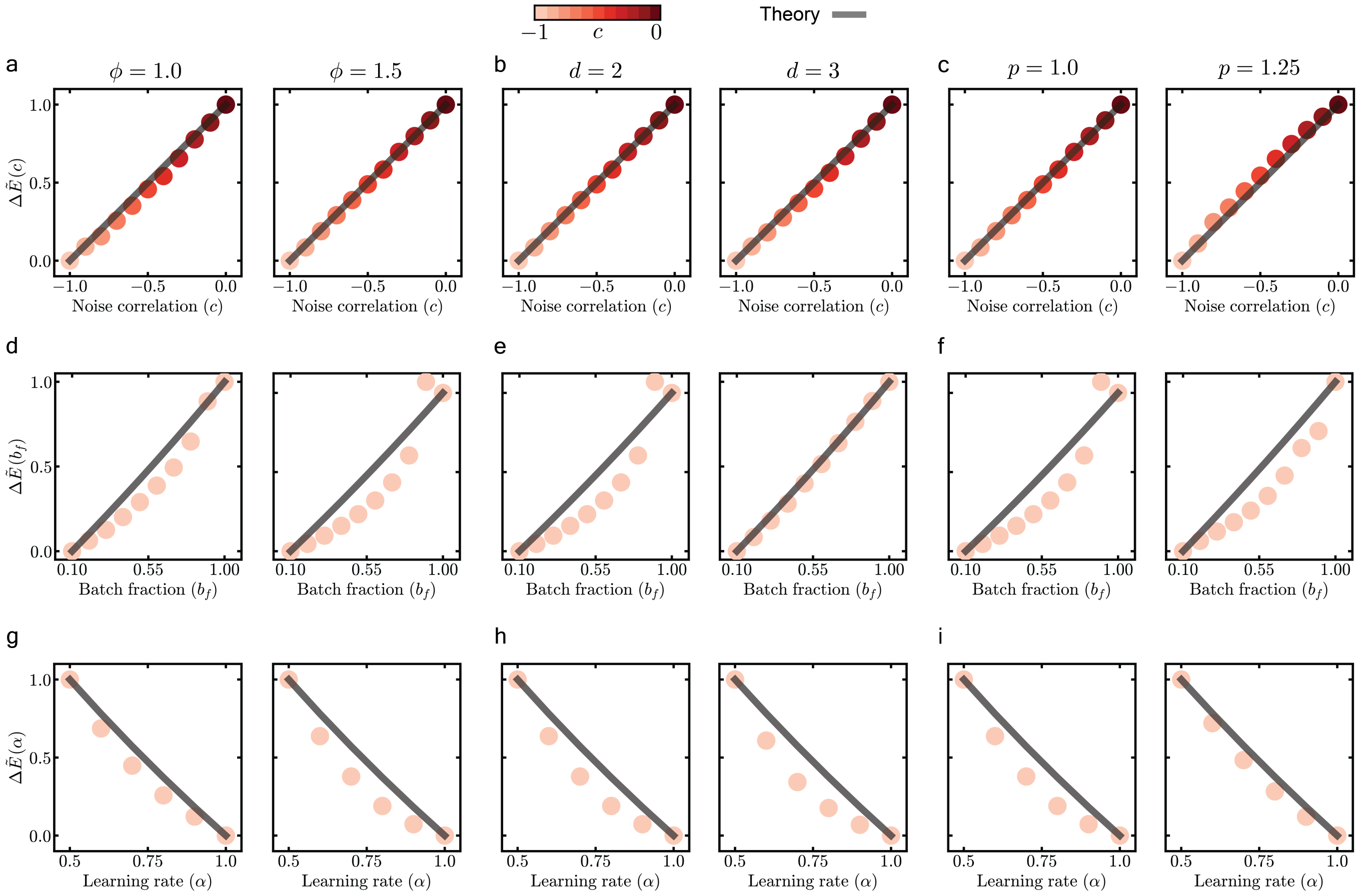

FIG. S5. Dependence of flatness of energy landscape on system parameters for Stochastic Gradient Descent (SGD). Normalized energy change $\Delta\tilde{E}(c)$ versus noise correlation c for different particle volume fraction ϕ (a), spatial dimension d (b), and stiffness of the potential p (Eq. S11) (c). $\Delta E(c)$ is normalized as: $\Delta\tilde{E}(c) = [\Delta E(c) - \Delta E(c = -1)]/[\Delta E(c = 0) - \Delta E(c = -1)]$. Black lines show prediction of Eq. S79 (SI Sec. I.C). Normalized energy change $\Delta\tilde{E}(b_f)$ versus batch fraction b_f for different particle volume fraction ϕ (d), spatial dimension d (e), and stiffness of the potential p (Eq. S11) (f). $\Delta E(b_f)$ is normalized as: $\Delta\tilde{E}(b_f) = [\Delta E(b_f) - \Delta E(b_f = 0.1)]/[\Delta E(b_f = 1.0) - \Delta E(b_f = 0.1)]$. Black lines show prediction of Eq. S82 (SI Sec. I.C). Normalized energy change $\Delta\tilde{E}(\alpha)$ versus learning rate α for different particle volume fraction ϕ (a), spatial dimension d (b), and stiffness of the potential p (Eq. S11) (c). $\Delta E(\alpha)$ is normalized as: $\Delta E(\alpha) = [\Delta E(\alpha) - \Delta E(\alpha = 1.0)]/[\Delta E(\alpha = 0.5) - \Delta E(\alpha = 1.0)]$. Black lines show prediction of Eq. S81 (SI Sec. I.C). The baseline parameters are set to $N = 100000$, $\alpha = 0.25$, $\phi = 1.5$, $d = 2$, $b_f = 0.5$, $p = 1.0$, and $c = -1$, with each panel varying a single parameter. For $d = 3$, however, we use $\phi = 0.75$ instead, as higher volume fractions such as $\phi = 1.5$ result in very small values of ΔE, making it difficult to measure accurately in simulations. All symbols denote discrete-time particle simulations.

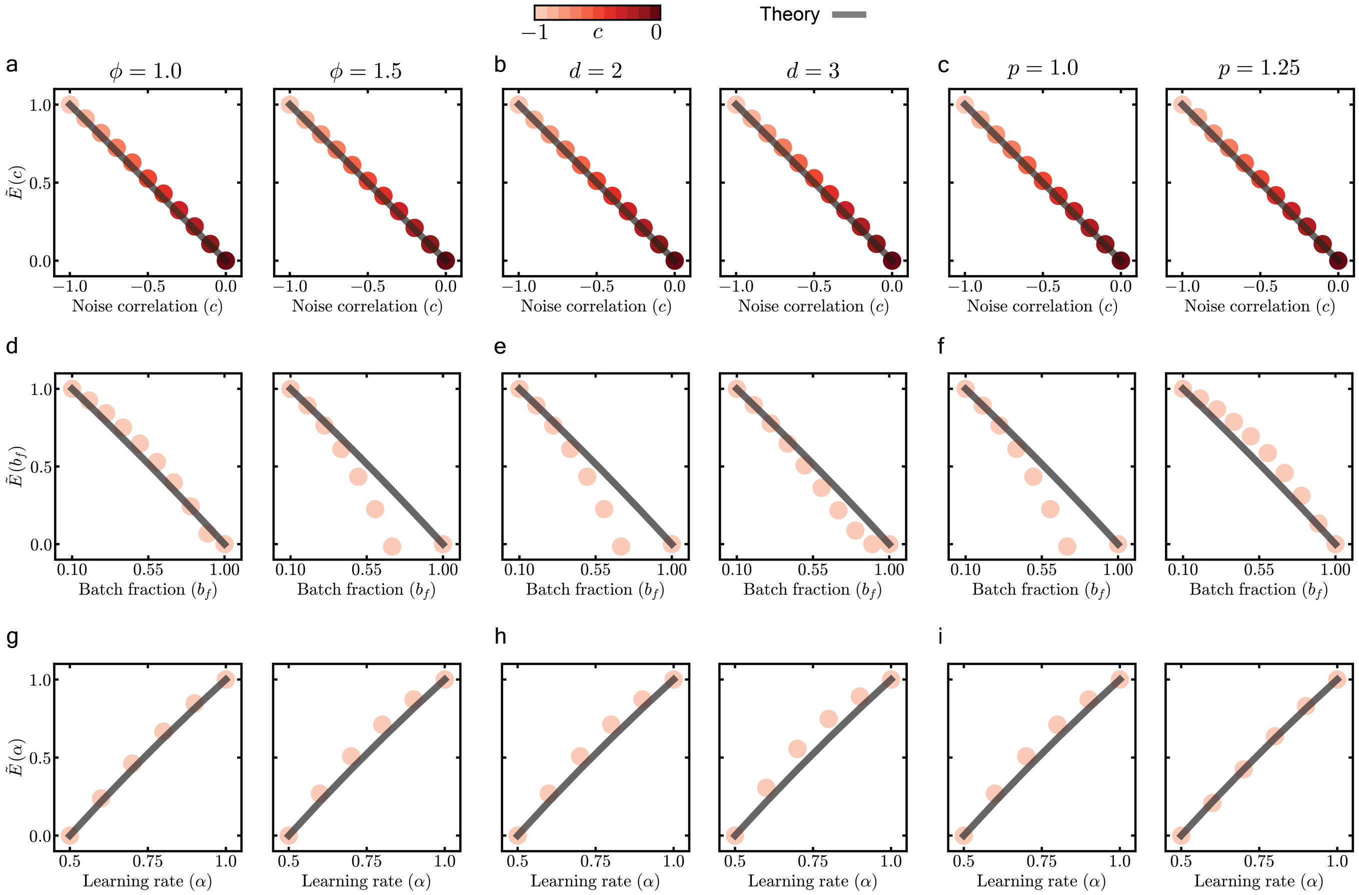

FIG. S6. Dependence of energy of the steady-state configuration on system parameters for Stochastic Gradient Descent (SGD). Normalized energy $\tilde{E}(c)$ versus noise correlation c for different particle volume fraction ϕ (a), spatial dimension d (b), and stiffness of the potential p (Eq. S11) (c). $E(c)$ is normalized as: $\tilde{E}(c) = [E(c) - E(c = 0)]/[E(c = -1) - E(c = 0)]$. Black lines show prediction of Eq. S72 (SI Sec. I.C). Normalized energy $\tilde{E}(b_f)$ versus batch fraction b_f for different particle volume fraction ϕ (d), spatial dimension d (e), and stiffness of the potential p (Eq. S11) (f). $E(b_f)$ is normalized as: $\tilde{E}(b_f) = [E(b_f) - E(b_f = 1.0)]/[E(b_f = 0.1) - E(b_f = 1.0)]$. Black lines show prediction of Eq. S75 (SI Sec. I.C). Normalized energy change $\tilde{E}(\alpha)$ versus learning rate α for different particle volume fraction ϕ (a), spatial dimension d (b), and stiffness of the potential p (Eq. S11) (c). $E(\alpha)$ is normalized as: $\tilde{E}(\alpha) = [E(\alpha) - E(\alpha = 0.5)]/[E(\alpha = 1.0) - E(\alpha = 0.5)]$. Black lines show prediction of Eq. S74 (SI Sec. I.C). All parameters are the same as Fig. S5 (see caption). All symbols denote discrete-time particle simulations.

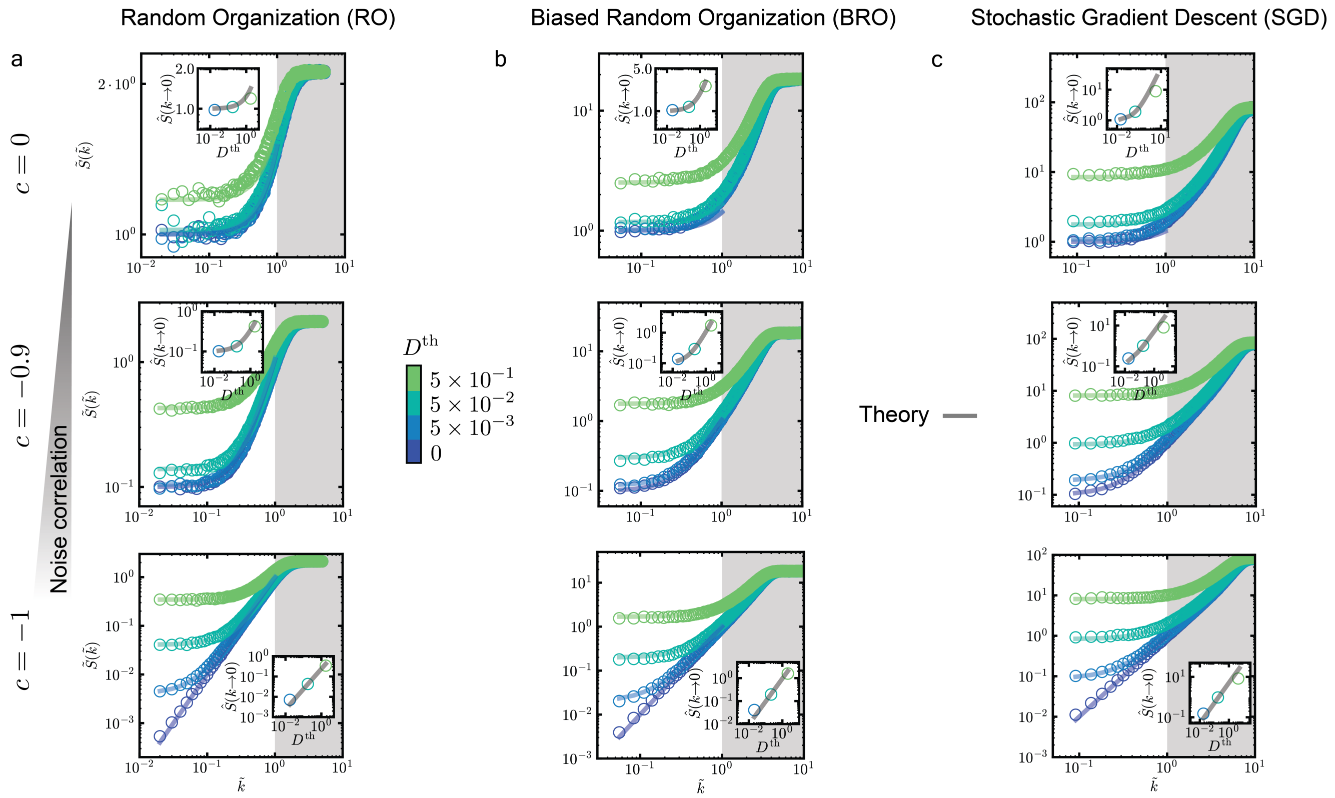

FIG. S7. Long-range structure in the presence of thermal noise in random-organizing systems. Normalized radially averaged structure factor $\tilde{S}(\tilde{k})$ versus normalized radial wave number $\tilde{k}$ for random-organization (RO) (a), biased random-organization (BRO) (b), and stochastic gradient descent (SGD) (c) in discrete-time particle simulations. All parameters are the same as Fig. 2 in main text (see Methods). $\tilde{S} = S(k)/S_0(2\pi/L)$ where $S_0(2\pi/L)$ is the structure factor for $c = 0$ at $k = 2\pi/L$, and L is the side length of the simulation box. $\tilde{k} = k/k_0$ where k_0 is the value at which $\tilde{S}(k_0) = 1$ for anti-correlated noise ($c = -1$) of the same system. The solid colored lines show a combined best fit of Eq. S85 to various noise correlations c for each system, using D^{th} as the single fitting parameter. Insets show $\hat{S}(\tilde{k} \to 0)$ versus thermal diffusion coefficient D^{th}. $\hat{S}(\tilde{k} \to 0, c, D^{\mathrm{th}}) = \tilde{S}(\tilde{k} \to 0, c, D^{\mathrm{th}})/\tilde{S}(\tilde{k} \to 0, c = 0, D^{\mathrm{th}} = 0)$. Solid black lines in the insets denote $1 + c + 2D^{\mathrm{th}}/\bar{\rho}A_1$ (prediction of Eq. S85 in the limit $\tilde{k} \to 0$). Gray shaded regions denote short-range behavior ($\tilde{k} > 1$).

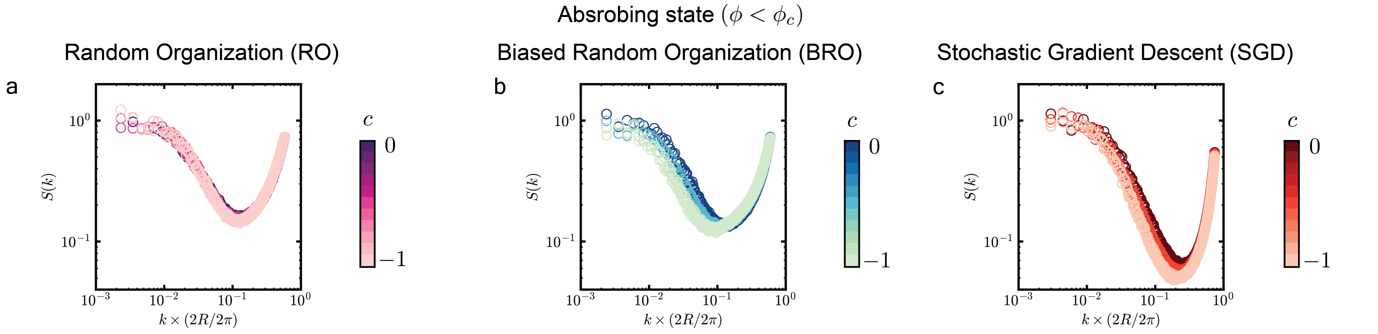

FIG. S8. Long-range structure in the absorbing phase ($\phi < \phi_c$) of random-organizing systems, where ϕ_c denotes the critical particle volume fraction. Radially averaged structure factor $S(k)$ versus dimensionless radial wave number $k \times (2R/2\pi)$ in discrete-time particle simulations, where R is the particle radius, for (a) random-organization (RO); (b) biased random-organization (BRO); and (c) stochastic gradient descent (SGD). All parameters are the same as in the main text (see Methods), except ϕ, which is set to $\phi/\phi_c = 0.9$ for all systems.
